# Localized high-risk prostate cancer harbors an androgen receptor activity–low subpopulation susceptible to HER2 inhibition

**DOI:** 10.1172/JCI189900

**Published:** 2025-09-04

**Authors:** Scott Wilkinson, Anson T. Ku, Rosina T. Lis, Isaiah M. King, Daniel Low, Shana Y. Trostel, John R. Bright, Nicholas T. Terrigino, Anna Baj, Emily R. Summerbell, Kayla E. Heyward, Sumeyra Kartal, John M. Fenimore, Chennan Li, Cassandra Singler, BaoHan Vo, Caroline S. Jansen, Huihui Ye, Nichelle C. Whitlock, Stephanie A. Harmon, Nicole V. Carrabba, Rayann Atway, Ross Lake, David Y. Takeda, Haydn T. Kissick, Peter A. Pinto, Peter L. Choyke, Baris Turkbey, William L. Dahut, Fatima Karzai, Adam G. Sowalsky

**Affiliations:** 1 Genitourinary Malignancies Branch, National Cancer Institute, Bethesda, Maryland, USA.; 2 Office of Intramural Research, National Institutes of Health, Bethesda, Maryland, USA.; 3 Radiation Oncology Branch, National Cancer Institute, Bethesda, Maryland, USA.; 4 Department of Urology, Emory University School of Medicine, Atlanta, Georgia, USA.; 5 Department of Pathology and Department of Urology, UCLA, Los Angeles, California, USA.; 6 Molecular Imaging Branch,; 7 Laboratory of Cancer Biology and Genetics, and; 8 Urologic Oncology Branch, National Cancer Institute, Bethesda, Maryland, USA.

**Keywords:** Genetics, Oncology, Molecular pathology, Oncogenes, Prostate cancer

## Abstract

**BACKGROUND:**

Localized high-risk prostate cancer (PCa) often recurs despite neoadjuvant androgen deprivation therapy (ADT). We sought to identify baseline molecular programs that predict pathologic response and reveal targetable vulnerabilities.

**METHODS:**

We profiled 147 biopsy foci from 48 MRI-visible lesions in 37 patients before 6 months of ADT plus enzalutamide and radical prostatectomy. Residual cancer burden (RCB) at prostatectomy was the primary outcome. Analyses incorporated PTEN loss, TMPRSS2:ERG status, and HER2/androgen receptor (AR) immunohistochemistry on baseline and posttreatment tissues. Findings were evaluated in an external transcriptional cohort (*n* = 121) and by multiplex immunostaining in an independent cohort (*n* = 61). Functional assays tested enzalutamide-responsive enhancers near *ERBB2* and sensitivity to HER2 inhibition.

**RESULTS:**

A baseline, HER2-associated transcriptional program correlated with higher RCB and inversely with AR activity, independent of PTEN and ERG. Exceptional responders had lower HER2 protein levels in pretreatment biopsy specimens. The inverse AR-HER2 relationship recurred across data sets and multiplex immunostaining, which revealed coexisting AR-high/HER2-low and HER2-high/AR-low subpopulations. Enzalutamide inhibited AR-mediated repression of *ERBB2*. HER2-high/AR-low cells present before therapy resisted ADT yet were sensitive to HER2 inhibitors; combining HER2 inhibitors with enzalutamide increased tumor cell killing. These findings were reproduced in the external cohort and orthogonal assays.

**CONCLUSION:**

Baseline HER2 activity marks intrinsic resistance to neoadjuvant ADT in localized high-risk PCa and identifies a preexisting, targetable AR-low subpopulation. HER2-directed therapy, alone or with AR blockade, warrants clinical evaluation.

**TRIAL REGISTRATION:**

ClinicalTrials.gov registration: NCT02430480.

**FUNDING:**

Prostate Cancer Foundation; Department of Defense Prostate Cancer Research Program; National Institutes of Health.

## Abstract 2

Aggressive untreated prostate cancers harbor a subpopulation of tumor cells with elevated levels of HER2 that may be sensitive to HER2 inhibitors

## Introduction

The molecular and clinical heterogeneities of localized high-risk (also known as locally advanced) prostate cancer (PCa) pose considerable obstacles for its diagnosis and treatment (1, 2). Although the use of CT and MRI has improved in vivo localization of extraprostatic disease that is associated with higher risk of prostate-specific antigen (PSA) or biochemical recurrence (BCR) or metastasis, substantial weight is given to the volume and extent of dedifferentiation of PCa visualized on needle biopsies for assessing individualized risk (3). Patients with locally advanced PCa receive multimodal therapies, which can include androgen deprivation therapy (ADT) with radiation therapy or adjuvant treatment following surgery (3–6). In patients treated by radical prostatectomy, adjuvant or neoadjuvant (before surgery) therapies reduce local tumor burden and target occult micrometastases that would eventually drive relapse (6, 7). Phase 2 trials of different neoadjuvant therapies for localized high-risk PCa have tested multiple combinations of hormonal and chemohormonal therapies, including second- and third-generation androgen receptor–targeting (AR-targeting) agents (8–13), cytotoxic chemotherapy (14–16), and immunotherapy (17–19). A persistent challenge common to all of these studies has been identifying robust baseline characteristics that would show long-term survival benefits based on prognostic features readily identified by short-term pathologic readouts.

In clinical trials of neoadjuvant intensive ADT, the biomarker showing the most robust performance toward predicting long-term outcome is residual cancer burden (RCB), which is calculated on the basis of the tumor cellularity of the final pathologic specimen. Patients with lower RCB having complete pathologic responses (pCRs) or minimal residual disease (MRD) demonstrated delayed BCR (20, 21), with 3-year BCR-free rates of 95.2% (pCR/MRD) versus 48.7% (greater than MRD). Using RCB or BCR as end points, molecular features detected in both posttreatment and baseline tissues have been reported with various prognostic potential, including the mutation status of *TP53* (22–25) and IHC against PTEN or ERG (9, 10, 22). Across all reported studies, nearly every patient has shown at least some pathologic and/or imaging response to therapy. What remains unknown, however, is the extent to which clonal or subclonal intrinsic resistance to neoadjuvant intensive ADT, even in patients showing partial responses, can be targeted further.

Intrinsic resistance to targeted therapies involves cell-intrinsic mechanisms implicating the original target, which, in the case of PCa, is the AR (26, 27). In contrast to well-documented mechanisms of adaptive resistance involving AR point mutations or amplification after long-term exposure to ADT (27–29), a subset of aggressive prostate tumors displays resistance to ADT as measured by transcriptional profiling of the tumor to have lower AR activity (24, 30, 31). Due to the challenges of extensive profiling of biopsy tissues, genomic, histologic, and phenotypic properties of these “low-AR” tumors are not well characterized. Cellular mechanisms that suppress AR activity, or that may otherwise be elevated in the absence of AR activity, have similarly remained elusive. Limited histologic and transcriptomic profiling of prostatectomy tissues after neoadjuvant ADT has identified potentially targetable “bypass” pathways, including AKT, HER2/HER3, CDK4/6, MET, BCL-2, and XBP1 (25, 32, 33). Although the detection of phosphorylated HER2 (pHER2) in metastatic PCa tumor biopsies supported previous clinical studies and was proposed as an adaptive mechanism to prolonged ADT exposure, it showed little clinical benefit in this context (32, 34–37). Here, we present HER2 activity not as an adaptive bypass pathway but as an inherent and targetable mechanism of resistance in de novo locally advanced PCa with AR activity–low characteristics.

## Results

### Prostate tumors exhibiting poor pathologic response to neoadjuvant ADT plus enzalutamide harbor a transcriptional signature of elevated HER2 activity.

**Figure 1:**
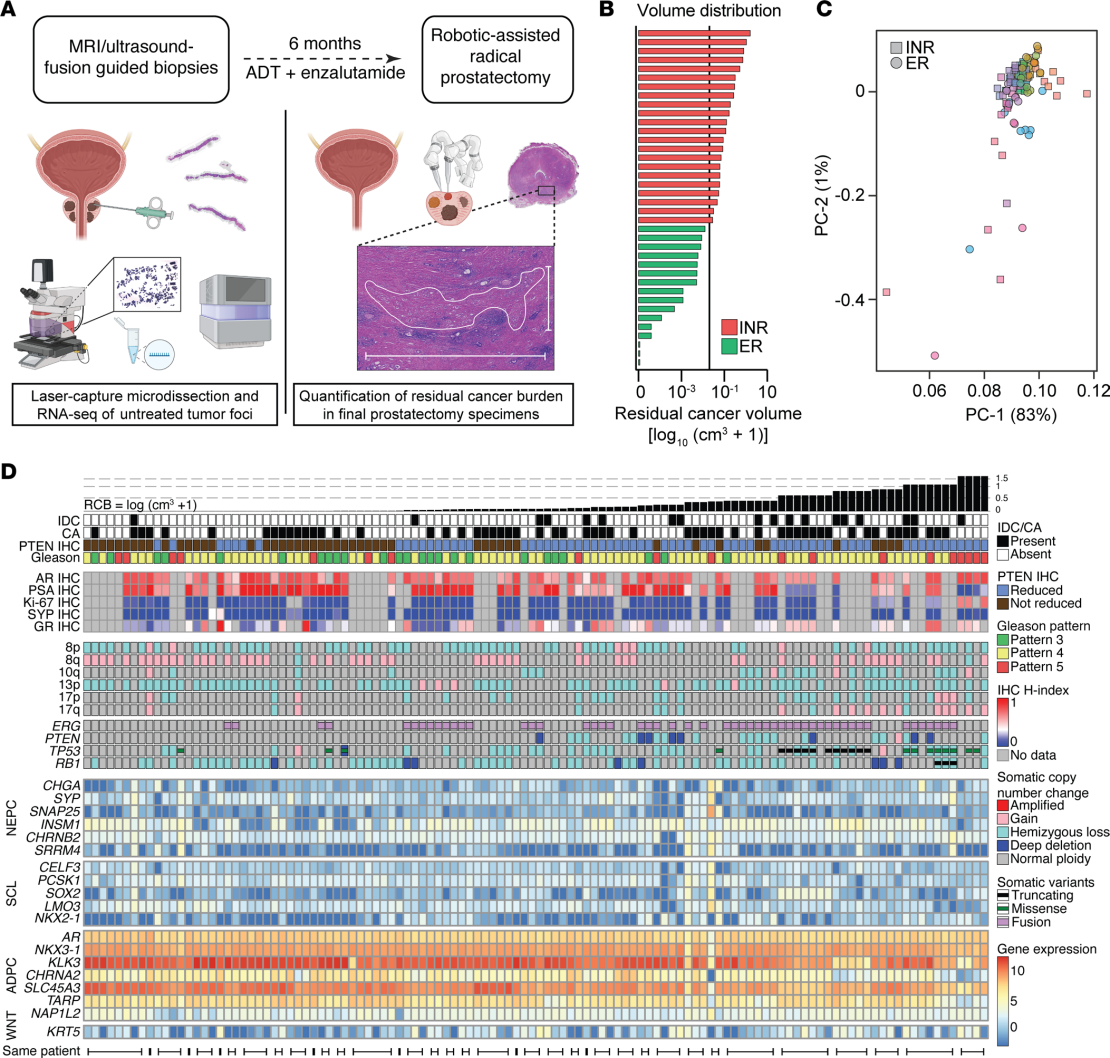
Integrated molecular landscape of prostate tumors prior to neoadjuvant-intense ADT. (**A**) Schematic of workflow in which LCM and RNA-Seq of tumor foci from image-guided baseline biopsy specimens (left) were used to assess gene expression differences that track with posttreatment pathologic tumor volumes (right). (**B**) Distribution of RCB (1 row per patient) plotted on a logarithmic *x*-axis with a pseudocount (cm^3^ + 1). ER, exceptional responders (residual tumor volumes <0.05 cm^3^). INR patients had residual tumor volumes ≥0.05 cm^3^. (**C**) Principal component (PC) analysis of 147 baseline tumor foci transcriptomes. Each dot is colored by patient, with squares representing foci from INR patients and circles representing foci from ER patients. (**D**) Heatmap and oncoprint depicting molecular and histologic features of baseline tumors. Each column represents 1 laser-capture microdissected tumor focus subjected to whole-transcriptome sequencing. Identical values are given to IHC profiling performed on a single tissue that was subdivided for sequencing. Black bars at the bottom indicate multiple samples from the same patient. Samples are ranked from left to right by patient-level RCB volumes. Heatmap of IHC depicts histology intensity scores reported by Wilkinson et al. (22). ADPC, adenocarcinoma; NEPC, neuroendocrine; SCL, stem cell-like; WNT, Wnt-dependent.

Using whole-transcriptome sequencing, we assessed patterns of gene expression and pathway activation in 147 tumor foci isolated from prostate biopsy specimens prior to the patient undergoing 6 months of intense neoadjuvant ADT (Figure 1A). These foci were sampled using laser capture microdissection (LCM) from 48 distinct, MRI-visible lesions in 37 patients who participated in our clinical trial, with a median of 2 biopsy blocks per patient and a median of 2 foci per block sequenced. Most foci were isolated separately on the basis of differing histologic features, such as Gleason pattern or variability in key PCa markers such as PTEN or ERG staining.

Our overall goal was to identify variability in genes and pathways expressed at baseline that would track with resistance to therapy. Therefore, we initially stratified samples by our predefined cut point of 0.05 cm^3^ posttreatment residual tumor volume, which was measured from the largest area of tumor in the final prostatectomy specimens (Figure 1, A and B, and see Methods) (8). Because we also sequenced foci from resolving MRI lesions that were acquired from nonresponding patients, we further limited our analysis to 117 foci isolated only from MRI index lesions. By principal component analysis, however, the samples did not form distinct groupings separating exceptional responder patients versus incomplete and nonresponder (INR) patients (Figure 1C). In addition, application of 4 predefined gene groups that had previously been shown to segregate treatment-resistant tumors into transcriptionally defined subtypes in the castration-resistant setting (38) did not prominently identify multiple baseline tumor subgroups at the transcriptomic level (Figure 1D). Rather, nearly every focus showed strong enrichment for genes in the AR-dependent adenocarcinoma gene group, which included *AR*, *NKX3-1*, and *KLK3* (Figure 1D).

Nevertheless, we observed that tumors with larger RCB, located toward the right of the heatmap in Figure 1D, displayed lower expression of AR-dependent adenocarcinoma genes, including *KLK3*. To connect these gene expression observations to protein levels, we used PSA histology intensity scores we previously generated by automated IHC on this same cohort of samples (22). We found an inverse correlation with RCB on a per-patient basis (ρ = −0.43, *P*_ρ_ = 0.016).

**Figure 2:**
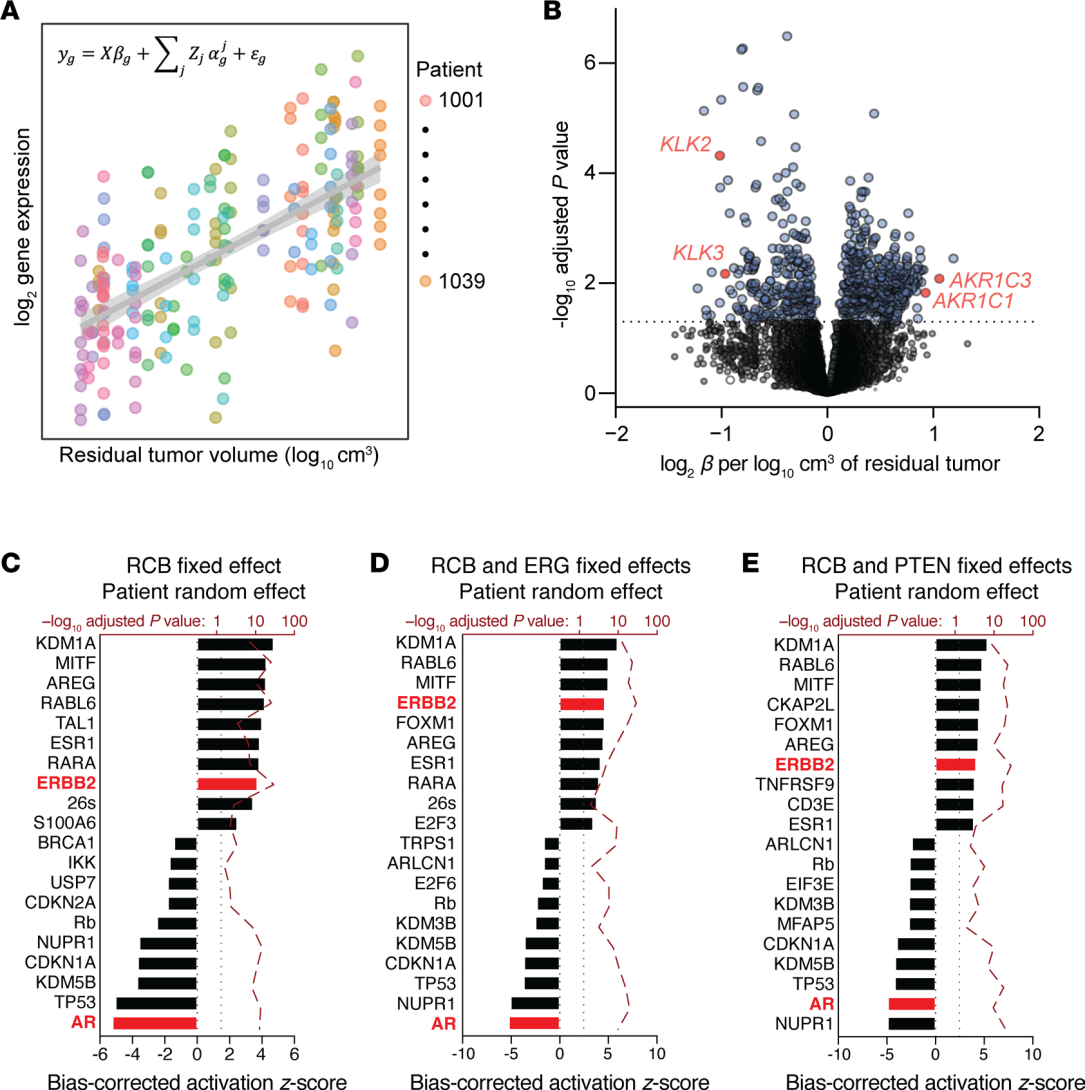
Pathologic response to neoadjuvant ADT plus enzalutamide is associated with a transcriptional signature of elevated HER2 activity at baseline. (**A**) Linear mixed-effects model depicting variance in gene expression across samples within each patient (by color) versus RCB (*x-*axis), showing gene expression patterns for positively correlating genes. (**B**) Volcano plot depicting DEGs determined using a linear mixed-effects model with RCB as a fixed effect and each patient as a random effect. Horizontal boundary depicts the *P*_adj_ = 0.05 cutoff. DEGs are quantified per cubic centimeter of posttreatment tumor volume: genes to the right are more expressed at baseline in tumors with higher posttreatment volumes, and genes to the left are less expressed. (**C**–**E**) All statistically significant DEGs (*P*_adj_ < 0.05) from the linear mixed-effects model were processed with the Upstream Regulator module of IPA. The 10 most activated and inactivated pathways (with *P*_adj_ < 0.05) are shown for DEG analyses in which (**C**) RCB was the only fixed effect, (**D**) RCB and ERG status were fixed effects, and (**E**) RCB and PTEN status were fixed effects. The bias-corrected *z* score is shown on the bottom *x*-axis and the *P*_adj_ value is shown on the top *x*-axis (–log_10_ transformed).

Therefore, we used RCB as a continuous variable to identify differentially expressed genes (DEGs) across the cohort. Retaining the variability afforded by multiply sampled cases, we used a linear mixed-effects statistical model, holding RCB as a fixed effect and modeling each patient as a random effect (Figure 2A). The outcome of this analysis identified 644 DEGs (adjusted *P* value [*P*_adj_] < 0.05), where the log_2_ *β* coefficient of the fixed effect is given as a magnitude of each gene’s relationship with residual tumor volume (unit per log cm^3^ of posttreatment RCB) (Figure 2B). Among the genes most negatively associated with posttreatment volume were the AR-responsive genes *KLK2* and *KLK3*, suggesting that tumors expressing higher levels of these genes at baseline had greater AR activity. By contrast, 2 of the most positive genes associated with posttreatment tumor volumes were *AKR1C1* and *AKR1C3* of the testosterone biosynthesis pathway, representing, in part, an adaptive response to low androgen levels in more aggressive prostate tumors (39).

To identify targetable pathways overrepresented in baseline tumors that resist therapy, we processed the 644-gene list using the Upstream Regulator module of Ingenuity Pathway Analysis (IPA). As depicted in Figure 2C, the most negatively enriched (inactivated) regulator was AR (*z* value for correlation [*z*_corr_] = ^–^5.2, *P*_adj_ = 2.6 × 10^–15^), and the upstream regulator with the lowest adjusted *P* value was *ERBB2* (human epidermal growth factor receptor 2 [HER2]) (*z*_corr_ = 3.7, *P*_adj_ = 2.6 × 10^–37^). Elevated HER2 activity, a targeted driver of many solid tumors including lung and breast, arises from *ERBB2* mutations, (co-)receptor amplification, or increased ligand binding (40). Given that we did not observe *ERBB2* mutations or amplification genomically in our cohort (22), we sought to validate this finding using differential gene expression as a function of known routes of pathway activation in vitro. We first identified 2 published data sets (41, 42) of HER2^+^ (i.e., *ERBB2-*overexpressing) breast cancer cell lines that were treated with growth factors (Supplemental Figure 1A; supplemental material available online with this article; https://doi.org/10.1172/JCI189900DS1). Both SKBR3 and BT-474 cells demonstrated activation of *ERBB2* using the Upstream Regulator module of IPA in response to stimulation with recombinant epidermal growth factor (EGF) versus a control, with BT-474 cells also showing a similar effect after stimulation with recombinant neuregulin 1 (NRG-1).

We next stimulated AR-positive LNCaP cells with 100 ng/mL EGF or NRG-1 for 5 minutes, 1 hour, and 4 hours. As anticipated, both EGF and NRG-1 induced acute phosphorylation of HER2 at Y1221/1222 at 5 minutes under both conditions, which persisted after 1 hour in NRG-1–stimulated cells (Supplemental Figure 1B), but not at 4 hours. By contrast, the 1 hour and 4 hour time points demonstrated strong activation of the Ingenuity *ERBB2* upstream regulator gene set (*z* score range: 1.8–2.1) (Supplemental Figure 1C), although gene expression–derived estimates of HER2 activity and direct phospho-protein detection of HER2 are not proportional as a function of time (Supplemental Figure 1D). Nonetheless, these data demonstrate that both routes of ligand activation of HER2 in PCa cells can drive elevation of the *ERBB2* transcriptomic signature.

As part of previous analyses, we found that both the *TMPRSS2-ERG* fusion (measured by either IHC or RNA-Seq) and *PTEN* loss (measured by IHC) were independently associated with poor pathologic responses (see Figure 1D) and had substantial effects on global transcription (22, 43). Therefore, we further modeled ERG or PTEN IHC status as additional fixed effects in our linear mixed-effects model. Regressing out the transcriptional impacts of ERG (Figure 2D) or PTEN (Figure 2E) did not appreciably change AR’s position or statistical significance in the bottom 10 inactivated pathways and HER2’s position in the top 10 activated pathways. Thus, HER2 activity represents a potential independent and distinctive mechanism of intrinsic resistance to neoadjuvant intense ADT.

### HER2 protein levels are associated with poor response.

**Figure 3:**
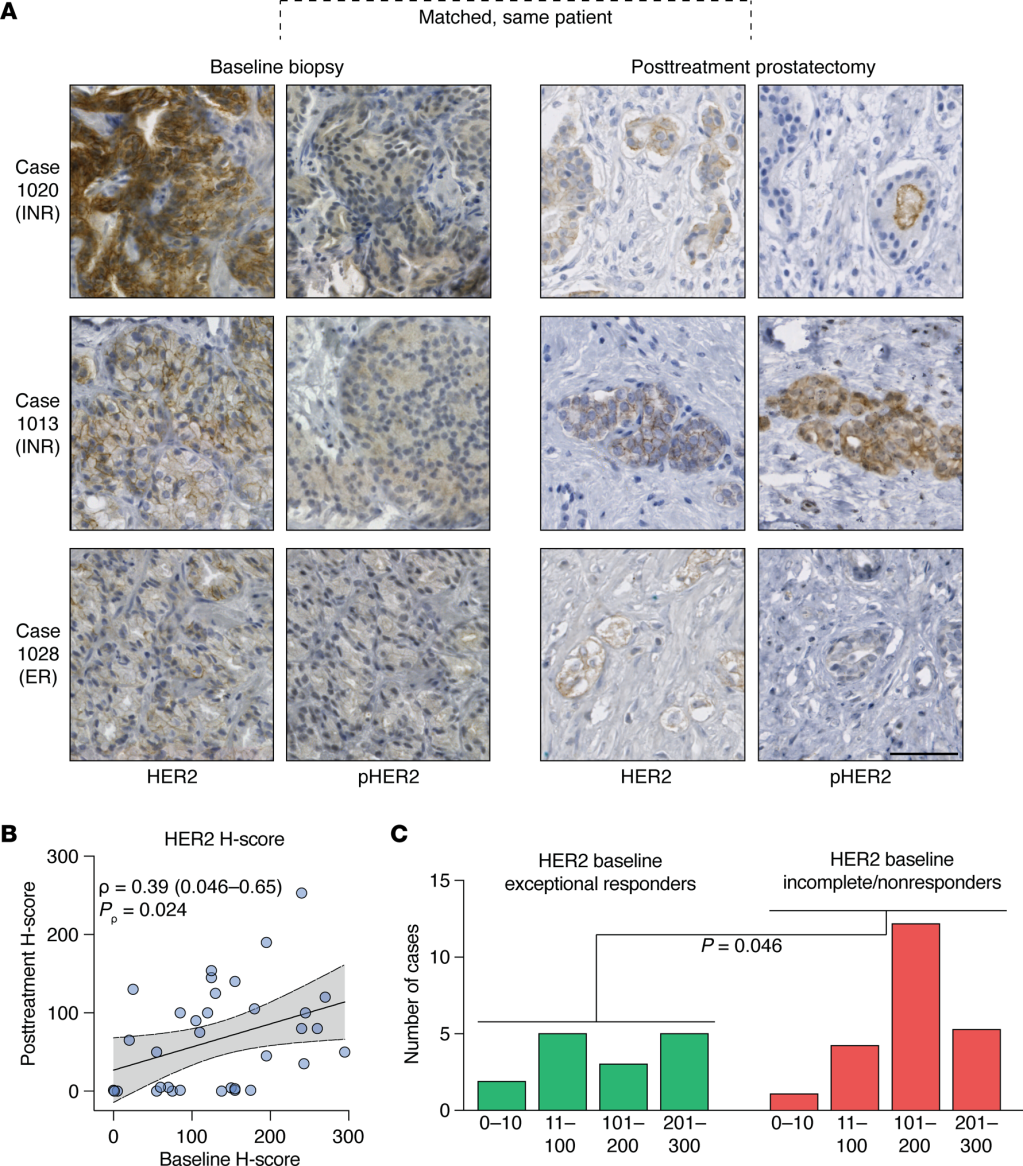
HER2 protein is expressed at baseline in tumor foci that resist therapy and is retained posttreatment. (**A**) Representative micrographs of anti-HER2 and anti-pHER2 IHC in baseline biopsies and residual tumor foci, showing examples from 3 patients with matched samples. Bar: 50 μm. (**B**) Scatter plot showing the association of per-patient HER2 baseline H-scores (*x*-axis) with posttreatment H-scores (*y*-axis). Statistical significance was determined using Spearman’s rank correlation. Line and gray shaded area show the linear regression line and 95% CI for the regression (0.046–0.65). (**C**) Density plots of HER2 baseline semi-quantitative IHC, stratified by pathologic response in the final surgical specimens. Statistical significance was determined by χ^2^ test.

*ERBB2*/HER2 up-regulation or activation has been implicated previously as an adaptive response to androgen deprivation and AR inhibition in metastatic PCa (35, 44) but not as a property of tumors intrinsically resistant to ADT. To confirm the results suggested by our transcriptional analyses, we performed a series of immunostains against total and pHER2 (Y1221/1222) in matched baseline biopsy specimens (*n* = 37 cases, 1–3 slides per case for HER2; *n* = 34 cases, 1 slide per case for pHER2) and posttreatment prostatectomies (*n* = 34 cases; 1–8 slides per case) (Figure 3A). Semi-quantitative H-scoring (low H-score: 11–100; medium H-score:101–200; and high H-score: 201–300) analysis by an expert genitourinary pathologist (RTL) was used to record separate cytosolic and membranous staining for HER2 (Supplemental Figure 2, A–C). Although HER2 at baseline was detected mostly in invasive tumor foci (both membranous and cytosolic), we separately scored intraductal foci, because they usually had greater levels of staining, using the maximum H-score for each case to compare trends across the cohort, irrespective of morphology, because the maximum H-score showed a positive correlation with measurements of *ERBB2* transcripts per million across 35 IHC-matched laser capture–microdissected foci (Supplemental Figure 2D). Although more cases were positive for HER2 at baseline (*n* = 34 of 37) (Supplemental Figure 2E) than posttreatment (*n* = 20 of 34) (Supplemental Figure 2F), we observed a statistically significant correlation between pre- and posttreatment IHC staining (by patient) for HER2 (ρ = 0.39, *P*_ρ_ = 0.024) (Figure 3B).

By contrast, pHER2 was detected in a minor fraction of all baseline and posttreatment foci (Supplemental Figure 3A). Most biopsy tissues (*n* = 30 of 31) and all prostatectomies (*n* = 80) displayed focal pHER2 expression at low or background (H-score: 0–10) levels (Supplemental Figure 3, B–D). Even though both HER2 and pHER2 levels were enriched at baseline in INR patients versus exceptional responder patients (*P* = 0.046 and *P* = 0.048, respectively, by χ^2^ test) (see Figure 3C and Supplemental Figure 3E, respectively), we cannot rule out preanalytical artifacts that interfered with pHER2 detection. Nonetheless, the absence of strong pHER2 raises the possibility that prostate tumors harbor HER2 activity in the absence of canonical membrane activation. Indeed, when we projected the transcriptomes of IHC-matched laser capture microdissected foci (*n* = 35) onto the DEGs from EGF and NRG-1 stimulation (see Supplemental Figure 1), we observed a statistically significant positive correlation with cytosolic but not membranous HER2 staining (Supplemental Figure 4, A–D).

We also explored the total and phosphorylated levels of EGFR and HER3, because persistent or constitutively active signaling through RTKs occurs in many solid tumor types including subtypes of breast cancer (HER2) and lung cancer (EGFR), especially when the gene is amplified. EGFR expression, but not its phosphorylation (Y1068), displayed similar patterns to HER2 staining (Supplemental Figure 5, A–E), although baseline expression stratified by pathologic outcome was not statistically significant (*P* = 0.10, by χ^2^ test) (Supplemental Figure 5F). HER3 and pHER3 (Y1289) expression was negligible across all samples (Supplemental Figure 5, G–I).

Overall, these findings are consistent with those in our prior report (22) of exome sequencing of tumor tissue from this study, which did not show any evidence of amplification or mutations in *EGFR*, *ERBB2*, or *ERBB3* that would result in high levels of membrane staining and/or constitutively-active receptor. However, these data confirm that an increase in HER2 protein levels at baseline is a molecular feature of tumors that go on to exhibit poor responses to neoadjuvant intense ADT plus enzalutamide, and that gene expression associated with steady-state cytosolic HER2 levels is consistent with gene signatures of persistent EGF and/or NRG-1 activation.

### PCa antiandrogen resistance is driven by a preexisting subpopulation with elevated HER2 activity.

**Figure 4:**
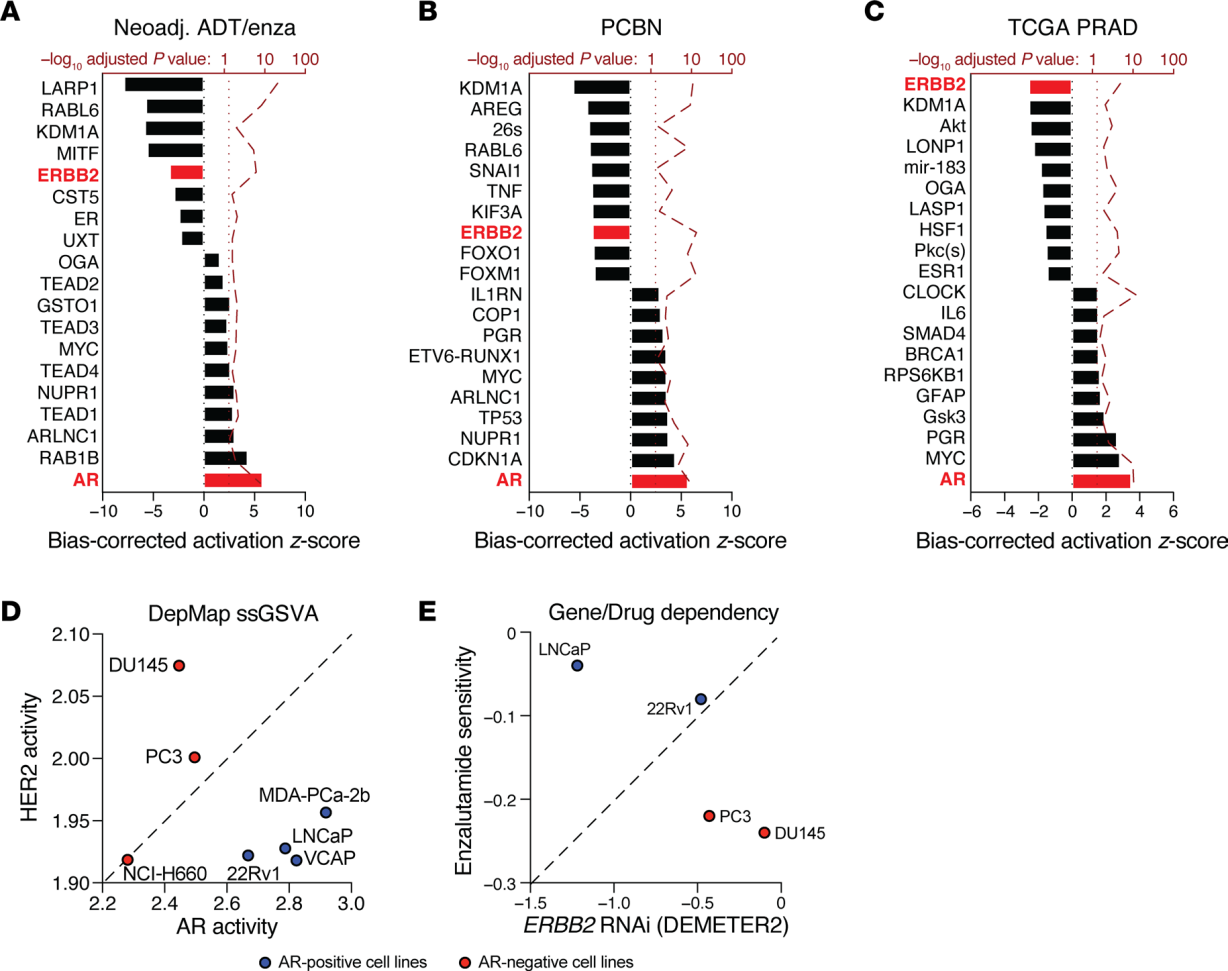
AR activity and HER2 activity maintain an inverse relationship in human prostate tumors and PCa cell lines. (**A**–**C**) Statistically significant DEGs (*P*_adj_ < 0.05) that were correlated with the “Hallmark_Androgen_Response” mSigDB gene set processed by single-sample GSVA (ssGSVA) were analyzed with the Upstream Regulator module of IPA for (**A**) our original neoadjuvant (neoadj) ADT plus enzalutamide (enza) cohort, (**B**) 123 tumors from the PCBN, and the (**C**) PCa cohort of The Cancer Genome Atlas (TCGA). PRAD, protatic adenocarcinoma. A linear mixed-effects model was used with the neoadjuvant cohort in (**A**) modeling repeated measures from patients as random effects. The 10 most activated and inactivated pathways (with *P*_adj_ < 0.05) are shown. (**D** and **E**) Publicly available data from the Broad Institute Dependency Map (DepMap) is shown, in which AR-positive cell lines are depicted in blue and AR-negative cell lines are depicted in red. Gene expression was summarized using ssGSVA for AR and HER2 activity signatures from mSigDB (**D**), and cell death (sensitivity) was plotted to compare matched enzalutamide sensitivity or *ERBB2* RNAi survival scores (**E**). DEMETER2 is an RNAi screen analytical framework within DepMap.

Because HER2 activity and its protein level at baseline tracked with pathologic outcome, we next sought to validate this finding. However, a lack of baseline samples acquired from other studies rendered this infeasible. Nonetheless, our observation that AR activity opposed HER2 activity offered an alternative approach amenable to any data set with whole-transcriptome gene expression. In particular, when we ranked our original data set by AR activity using single-sample gene set variation analysis of the Molecular Signatures Database (MSigDB) “Hallmark_Androgen_Response” gene set and examined those genes that tracked with AR activity, the Upstream Regulator module of IPA reported AR activity to be positively enriched (*z*_corr_ = 5.1, *P*_adj_ = 2.6 × 10^−8^) and *ERBB2* opposing it, now negatively enriched (*z*_corr_ = −2.4, *P*_adj_ = 8.4 × 10^−7^) (Figure 4A). We next used this approach on an independent cohort comprising 123 tumor samples we acquired from the Prostate Cancer Biorepository Network. We observed similar results (Figure 4B), with AR positively enriched as expected (*z*_corr_ = 5.7, *P*_adj_ = 3.0 × 10^−13^) and *ERBB2* negatively enriched (*z*_corr_ = –3.0, *P*_adj_ = 1.1 × 10^−15^). We also assessed this phenotype in the PCa cohort of The Cancer Genome Atlas, but due to concerns about substantial variability in purity arising from cell type admixtures from the original sample collection (45), we used deconvolution with dampened weighted least squares estimation to arrive at luminal PCa cell–specific gene expression values. AR was the most activated upstream regulator as expected (*z*_corr_ = 6.1, *P*_adj_ = 2.9 × 10^−9^) (Figure 4C), and *ERBB2* was again negatively enriched with the lowest adjusted *P* value (*z*_corr_ = –2.3, *P*_adj_ = 4.7 × 10^−5^). Collectively, these data suggest AR activity is inversely associated with HER2 activity across a range of untreated PCa cohorts.

Using data from the Broad Institute Dependency Map, we explored the relationship between AR or HER2 activity (similarly measured using single-sample gene set variation analysis), *ERBB2* expression, and enzalutamide sensitivity. AR activity stratified several established PCa cell lines, with NCI-H660, PC3, and DU145 cells having lower signature scores (Figure 4D). Two of these lines, DU145 and PC3 cells, also had higher HER2 scores, whereas the AR-high cell lines (22Rv1, VCaP, LNCaP, and MDA-PCa-2b cells) uniformly had lower HER2 scores. When comparing the 4 cell lines that had paired viability data from drug treatment (enzalutamide) and RNAi exposure (against *ERBB2*), LNCaP and 22Rv1 cells, which are AR-positive, had greater sensitivity to enzalutamide than did PC3 and DU145 cells (Figure 4E). However, PC3 and DU145 cells demonstrated impaired growth (relative to LNCaP cells) when treated with RNAi against *ERBB2*, indicating that established PCa cell lines may be acceptable models to explore the AR-HER2 relationship further, including the role of enzalutamide in regulating *ERBB2* expression and HER2 activity.

**Figure 5:**
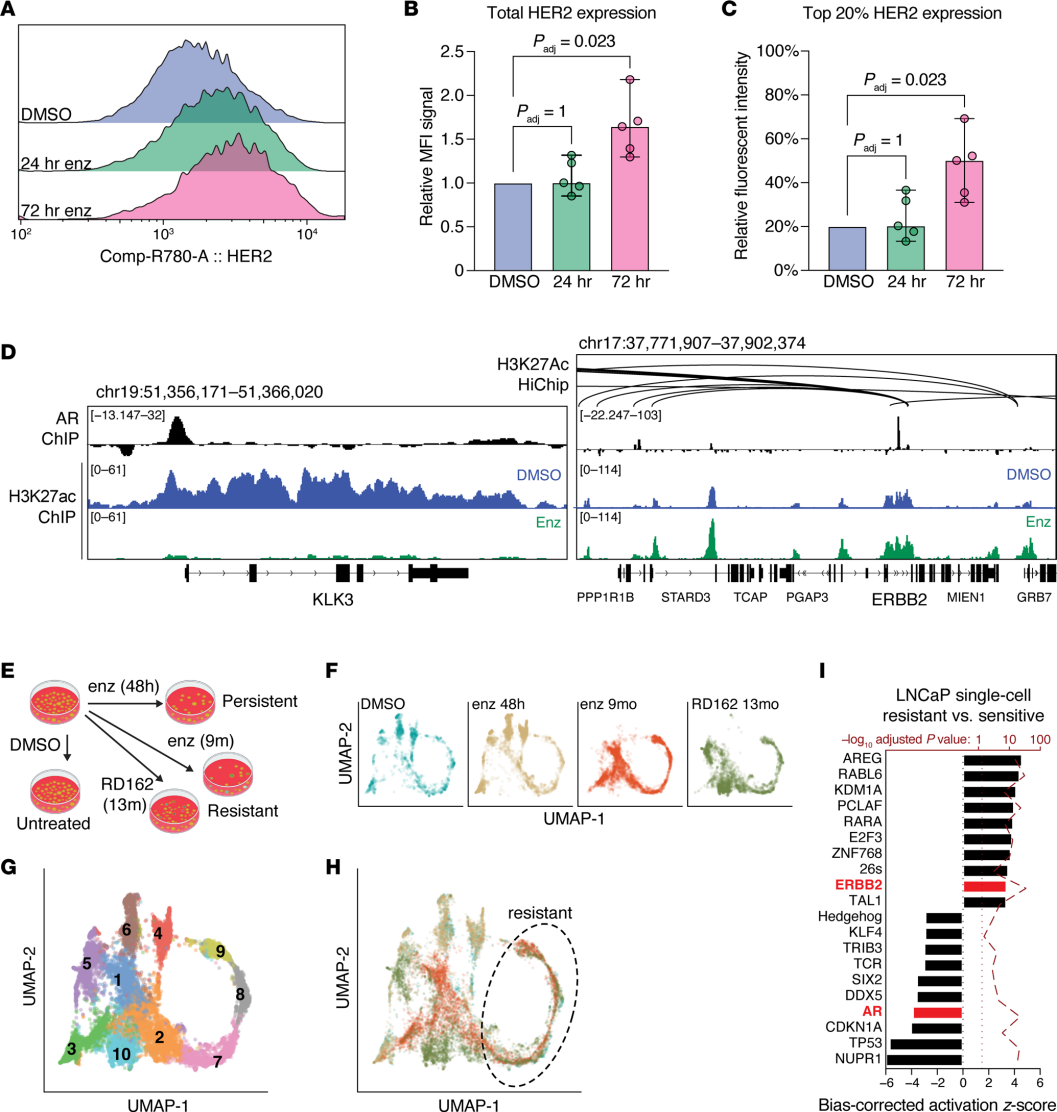
Enzalutamide treatment of prostate tumor cells selects for a preexisting subpopulation defined by low AR activity and high HER2 activity. (**A**) LNCaP cells were treated with enzalutamide (enz) for 0–3 days and subjected to flow cytometry with antibodies against HER2. HER2 fluorescent intensity is depicted as a histogram from a representative experiment. (**B** and **C**) Total HER2 (as MFI) (**B**) or the top 20th percentile of each individual experiment’s (**C**) control sample fluorescence was used as a cutoff for measuring subpopulations at the 24- and 72-hour time points. Lines indicate data medians; error bars are 95% CIs (*n* = 5). Statistical significance was determined by Friedman test with Dunn’s post hoc test. (**D**) AR ChIP-Seq, H3K27ac ChIP-Seq, and H3K27ac HiChIP profiles of LNCaP cells at the *KLK3* (left, shown as a control) and *ERBB2* (right) loci in enzalutamide or untreated (DMSO) conditions. (**E**–**I**) Single-cell gene expression data from LNCaP cells treated with antiandrogen (**E** and **F**) were downloaded and normalized together. (**F**–**H**) UMAP projections of each treatment condition individually (**F**), clustered by differential expression (**G**), and overlaid, colored by treatment condition (**H**). (**I**) Following trajectory and pseudobulk differential expression analysis, statistically significant DEGs (*P*_adj_ < 0.05) in the “resistant” clusters were analyzed with the Upstream Regulator module of IPA. The 10 most activated and inactivated pathways (with *P*_adj_ < 0.05) are shown.

To determine how inhibiting AR with enzalutamide affects HER2 levels, we first performed flow cytometry with anti-HER2 antibodies in LNCaP cells treated with enzalutamide over 3 days. As shown in Figure 5, A–C, inhibition of AR increased levels of HER2, enriching for the population of cells with higher levels of HER2, resulting in statistically significant increases after 72 hours by both MFI (*P*_adj_ = 0.023) and a subpopulation defined in each experiment as the top 20th percentile of HER2-expressing cells in the untreated control (*P*_adj_ = 0.023). To test whether this was due to direct effects of AR on the *ERBB2* locus, we performed anti-AR ChIP in LNCaP cells (Figure 5D) and identified a prominent binding peak in the middle of the *ERBB2* locus. We used previously published LNCaP H3K27ac ChIP and HiChIP data sets (46, 47), which indicated sites of potential enhancer looping between the AR binding site in *ERBB2* and proximal elements (within ~50 kb) that had increased H3K27 decoration upon enzalutamide treatment. This established that enzalutamide treatment selects for cells with transcriptional activation of *ERBB2* that is directly increased by AR inhibition.

To assess whether increased expression of *ERBB2* is an intrinsic property of these cells or an adaptive effect of AR inhibition, we accessed a published single-cell RNA-Seq data set (48) in which LNCaP cells were treated with antiandrogen and transcriptomically profiled shortly after exposure (48 hours) or after resistance developed (9–13 months) (Figure 5E). After joint normalization of the untreated (DMSO) control and treated samples, we used trajectory analysis to identify differentially expressed clusters of genes (Figure 5, F and G), with clusters 7, 8, and 9 best representing cell clusters present at baseline that were enriched upon resistance development (Figure 5H). We derived DEGs using pseudo-bulk analysis, comparing these 3 clusters with the rest. IPA recapitulated our earlier findings (Figure 5I), showing *ERBB2* activity increased in resistant cells (*z*_corr_ = 3.0, *P*_adj_ = 7.4 × 10^−38^), whereas AR activity decreased in sensitive cells (*z*_corr_ = −3.5, *P*_adj_ = 1.6 × 10^−24^). Collectively, these data suggest de novo PCa cell resistance to antiandrogen is driven, at least in part, by a preexisting subset of cells with elevated HER2 activity.

### AR-positive PCa cell lines are sensitive to HER2 inhibition, which enriches for cells with greater AR activity.

The increased abundance of HER2 activity in enzalutamide-resistant PCa cells raises the possibility that treatment with inhibitors against HER2 (or other RTK) may either increase tumor cell AR activity or enrich for the population of tumor cells that is harboring greater AR activity. We thus assessed the sensitivity of 8 different RTK inhibitors (RTKis)—afatinib (AFA), dacomitinib (DAC), erlotinib (ERL), gefitinib (GEF), lapatinib (LAP), neratinib (NER), sunitinib (SUN), and vandetanib (VAN)—against 3 different AR-positive PCa cell lines: LNCaP, LAPC-4, and 22Rv1 cells. LNCaP and LAPC4 cells represent an earlier state of hormone sensitivity in an untreated tumor, and 22Rv1 cells are AR-positive cells with treatment-acquired resistance to hormone therapy and antiandrogen.

**Figure 6:**
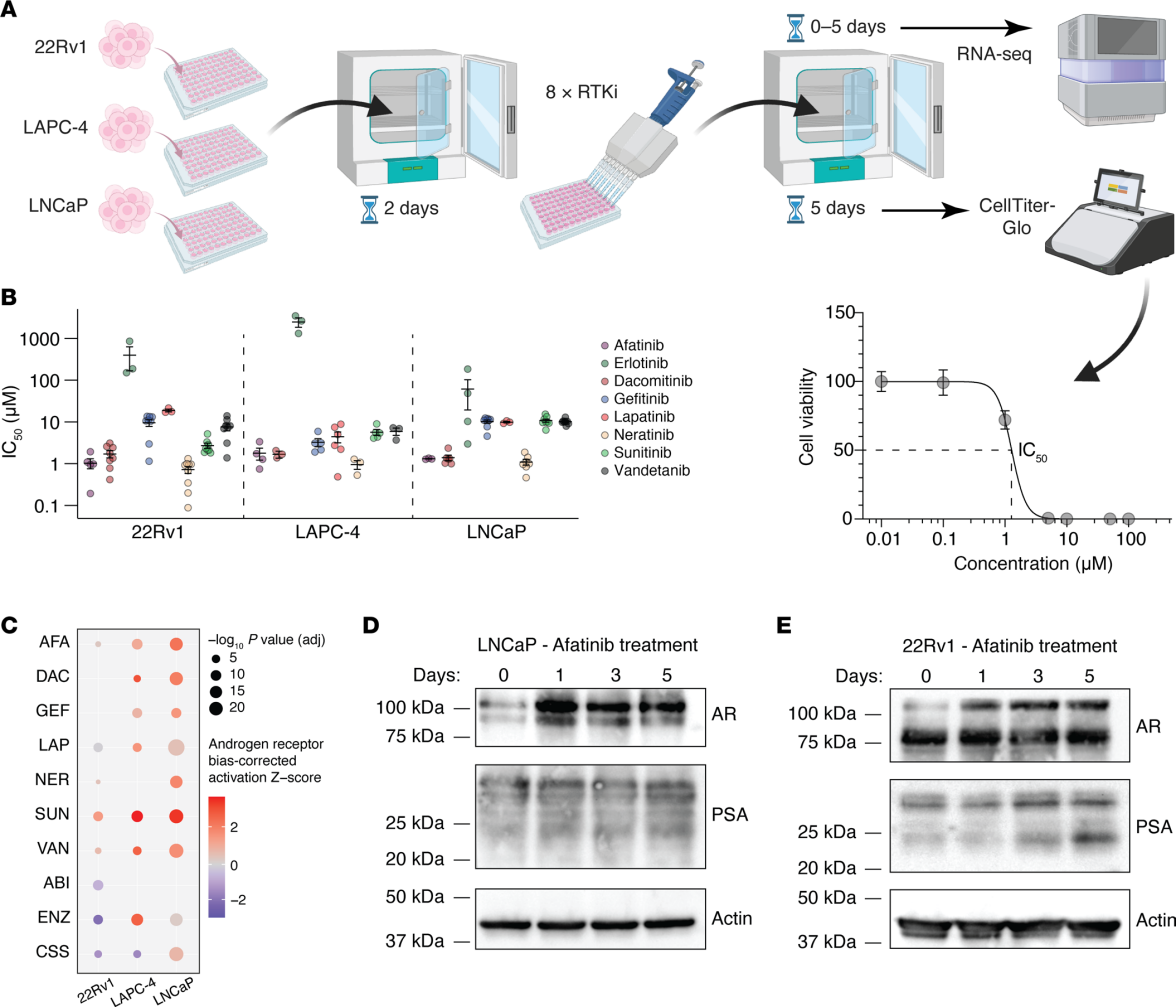
HER2 inhibition selects for PCa cells with greater AR activity. (**A**) Schematic depiction of in vitro screening of 22Rv1, LAPC-4, and LNCaP cells with 8 different receptor RTKis. (**B**) IC_50_ values are shown for individual lots of each RTKi used, per cell line. Lines and error bars depict the mean and SE data for at least 3 independent measurements. (**C**) Each cell line was treated with the indicated RTKi, abiraterone (ABI), or enzalutamide (ENZ) at the IC_50_ derived for that lot of drug. CSS was used in place of FBS (see Methods) in cell culture media. Treatments were performed over 5 days, and samples were acquired on days 0, 1, 3, and 5. RNA extracted from each sample (performed in duplicate) was subjected to whole-transcriptome sequencing, with DEGs (correlating with time) processed using the upstream regulator module of IPA. (**D** and **E**) Western blots depicting protein levels of LNCaP cells (**D**) and 22Rv1 cells (**E**) treated with AFA (at its empirically determined IC_50_) for 0–5 days. Blots shown are representative of at least 3 independent experiments. Actin is shown as a loading control.

Using a 7-concentration dose-response curve in sextuplicate, we derived IC_50_ values for each RTKi over 5 days, repeated at least 3 independent times (Figure 5, A and B), and assessed viability using CellTiter-Glo (CTG). As predicted, all 3 AR-positive cell lines displayed exquisite sensitivity to NER (range of IC_50_: 0.47–1.3 μM), an irreversible inhibitor selective for HER2. Cells were also sensitive to AFA (range of IC_50_: 1.0–2.8 μM), an inhibitor of homo- and heterodimerization for EGFR/HER2/HER3. Consistent with our finding that EGFR levels alone did not track with resistance (see Supplemental Figure 5), EGFR-specific inhibitors such as GEF and ERL displayed weak or no antitumor effect, respectively (Figure 6B).

Using these empirically determined IC_50_ doses, we next examined the impact of drug exposure on gene expression over 5 days across 3 independent experiments. As controls, we also treated cells with enzalutamide or abiraterone, or we grew cells with charcoal-stripped serum (CSS) to deplete the media of androgens. We extracted RNA at each time point and performed whole-transcriptome sequencing (Figure 6A). Using a linear mixed-effects model, we identified DEGs that changed in the same direction (up or down) over the entire time course, per unit time. We then processed these genes using the Upstream Regulator module of IPA to determine if AR activity was increasing on account of the RTK treatment (Figure 6C). Most RTKi treatments resulted in statistically significant and positive *z* scores for AR activity. Notably, we observed that upon treatment with AFA at each cell line’s IC_50_, increased AR pathway activity was observed across the LNCaP, LAPC-4, and 22Rv1 cell lines. Increases in AR activity were also observed with NER treatment for LNCaP and 22Rv1 cells, and surprisingly, treatment with the VEGFR inhibitor SUN also had a consistent and positive impact on AR activity. By contrast, treatment with ADT (CSS), abiraterone, or enzalutamide had mostly negative effects on AR activity.

At the protein level, treatment with AFA in both LNCaP (Figure 6D) and 22Rv1 (Figure 6E) cells dramatically enriched for AR expression after 1 day and persisted for up to 5 days. Increases in PSA protein levels were also observed over days 1–5 (Figure 6, D and E). Collectively, these findings suggest that RTKis, particularly the HER2 inhibitors AFA and NER, are highly potent on established AR-positive PCa cell lines, and that cells resistant to these treatments after 1–5 days express proportionally greater levels of AR.

### Nascent PCa harbors distinct HER2-high and AR activity–high subpopulations of tumor cells that are differentially responsive to HER2 inhibition.

**Figure 7:**
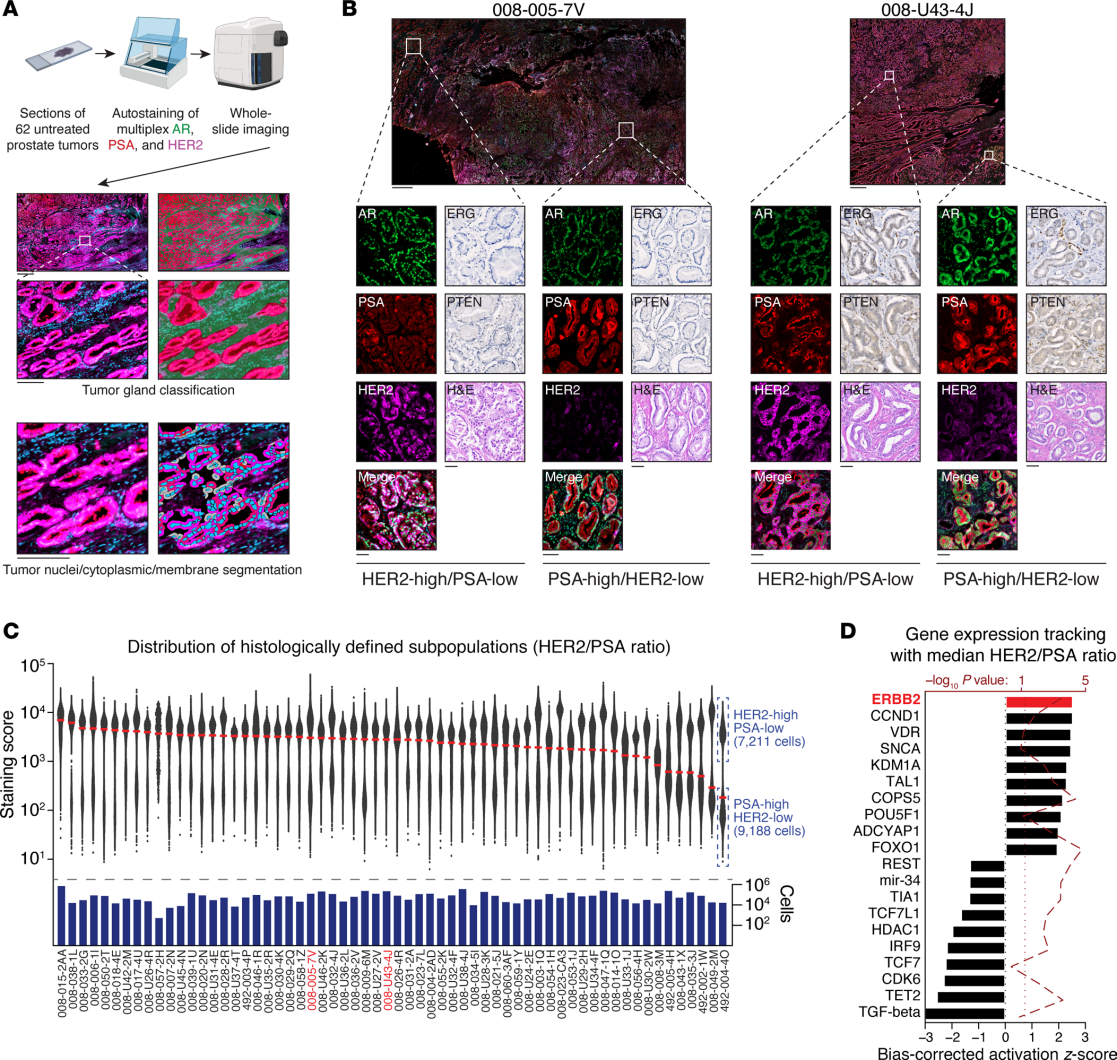
Human prostate tumors variably harbor distinct subpopulations of tumor cells with elevated levels of HER2 activity. (**A**) Schematic depiction of fully quantitative machine-guided analysis of multiplex staining. (**B**) Representative micrographs of AR activity–high/HER2 activity–low (HER2-low) and HER2 activity–high/AR activity–low tumor cell populations in 2 representative cases. Scale bar: 1 mm; inset bar: 100 μm. (**C**) Scatter plot representation of individual cellular HER2/PSA ratio scores for tumor cells in 62 slides, plotted on a log_10_ *y*-axis. Red bar represents median data. The number of fully segmented cells is shown below the scatter plot. The 2 cases indicated in red are the same cases depicted in **B**. Case 492-004-4O is enumerated to illustrate the distribution of cells. (**D**) Statistically significant DEGs (FDR < 0.1) that were correlated with the median HER2/PSA ratio for each case were analyzed with the Upstream Regulator module of IPA. The 10 most activated and inactivated biological pathways are shown.

We next investigated whether separate populations of AR activity–high and HER2-high cells coexist within the same prostate tumors, similar to what we have observed in vitro (see Figure 5). Therefore, we designed a multiplex immunofluorescent panel to capture nuclear AR, cellular PSA (as a readout of AR activity), and membrane-localized HER2 from FFPE sections of treatment-naive radical prostatectomy tissue. We applied this panel to 62 prostate tumors from 61 patients, performed whole-slide imaging, and analyzed the stained slides using the HALO AI platform to obtain tumor cell–specific segmented estimates of each marker’s expression (Figure 7A), covering a total of 5,655,686 segmented cells. We scored each cell using a ratio of HER2 membrane intensity to PSA cellular intensity (see Methods).

To our surprise, we found that having variable proportions of AR activity–high or HER2-high prostate tumor cells was a nearly universal feature of untreated (hormone-sensitive) localized prostate tumors. We observed distinct populations of PSA-high/HER2-low and HER2-high/PSA-low cells in most of these cases (Figure 7B), which were predominantly Gleason Grade Group 3. Although PCa luminal epithelial cells were universally AR-positive, regions that were PSA (and/or AR) activity–high were mutually exclusive to tumor foci that were expressing high levels of HER2, and vice versa. Consistent with our earlier analyses (see Figures 2, D and E), coexisting subpopulations were independent of concordant PTEN or *ERG*-fusion status (Figure 7B).

Although a bimodal distribution of these 2 phenotypes, as measured by HER2/PSA ratio score, was observed in most cases, the relative proportion of HER2-high versus AR activity–high cells varied across the cohort (Figure 7C). In some cases, a single homogeneous population was observed (Figure 7C). We performed bulk RNA-Seq on all 62 of these tumor sections and identified 466 DEGs (FDR < 0.1, log-ratio test) that tracked with the median immunofluorescence (IF) HER2/PSA ratio score across the cohort. When using the Upstream Regulator module of IPA, the most positively enriched (activated) regulator was *ERBB2* (*z*_corr_ = 2.2, *P* = 6.2 × 10^−3^), confirming that our HER2/PSA ratio score was accurately identifying tumors with elevated HER2 activity as also measured transcriptomically (Figure 7D).

**Figure 8:**
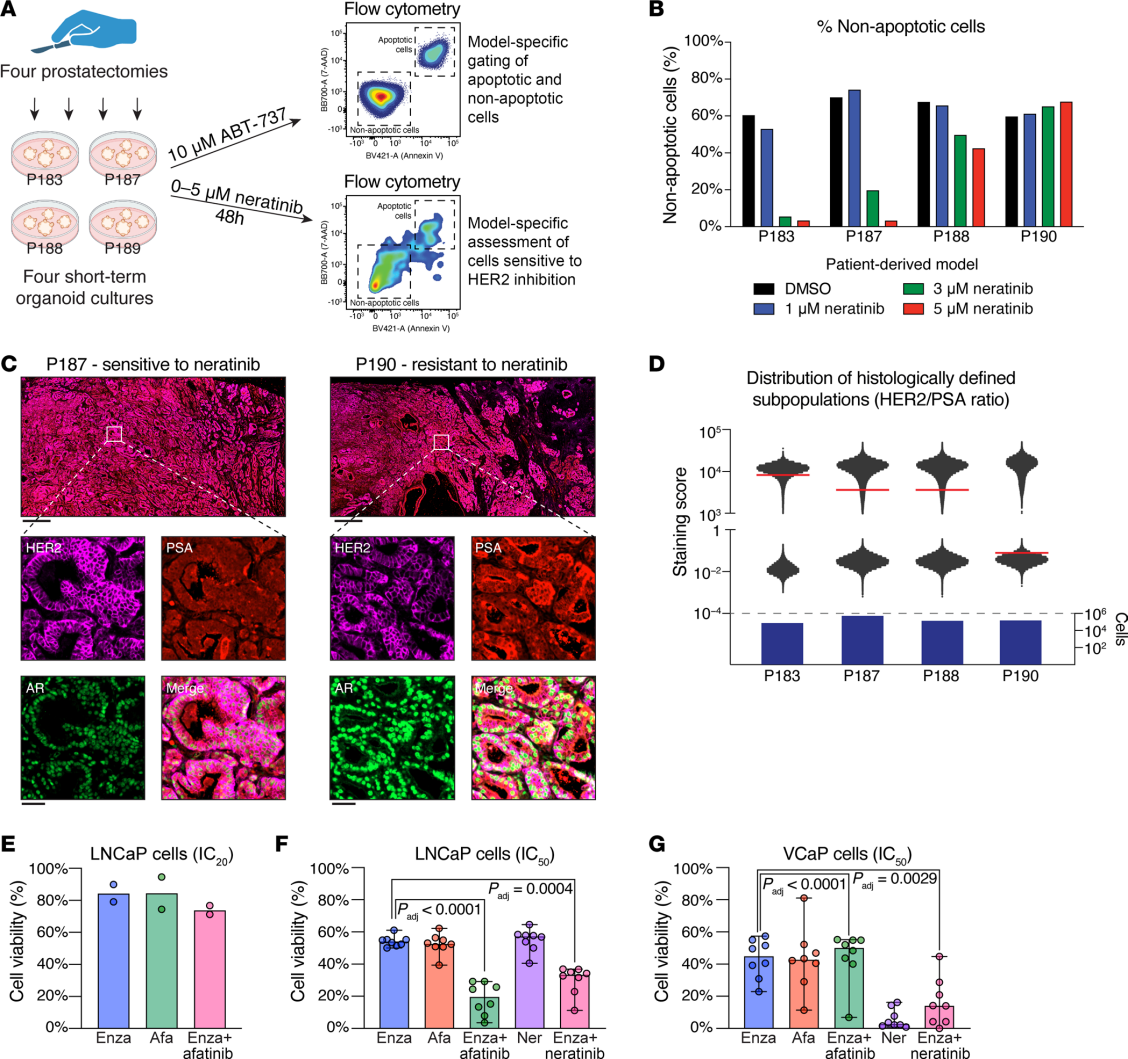
PCa cells expressing higher levels of HER2 are more sensitive to HER2 inhibition. (**A**) Schematic depiction of flow cytometry assays to measure tumor cell sensitivity to HER2 inhibition. (**B**) Bar graph shows the proportion of nonapoptotic cells measured in Supplemental Figure 7. (**C**) Representative micrographs of phenotypically dominant subpopulations from P187 and P190, showing HER2-high (P187; left) and PSA/AR-high subpopulations (P190; right), respectively. Uniform contrast enhancement was performed across both slides to enable direct visual comparison. Bar: 1 mm; inset bar: 100 μm. (**D**) Scatter plot representation of individual cellular HER2/PSA ratio scores of FFPE tumor sections from the radical prostatectomy specimens of tumors used to generate each organoid model, plotted on a log_10_ *y*-axis. Red bar represents median data. The number of fully segmented cells are shown below the scatter plot. (**E**) LNCaP cells were treated with either enzalutamide or AFA ± enzalutamide (at their respective IC_20_) for 5 days. Cell viability was measured using CTG. Data shown are the average of 2 experiments. (**F** and **G**) LNCaP (**F**) and VCaP (**G**) cells were treated with enzalutamide (Enza), AFA ± enzalutamide, or NER ± enzalutamide for 5 days. Cell viability was measured using CTG. Lines present median data; error bars represent 95% CIs (*n* = 8). Statistical significance was measured using a repeated-measures ANOVA test with Bonferroni adjustment for multiple comparisons.

Given the multiple lines of evidence supporting HER2 expression in human prostate tumors and the potency of HER2 inhibition in PCa cell lines (see Figure 6B), we next asked whether HER2 inhibition had an antitumor effect in human patient–derived PCa models. We established and treated 4 different PCa organoid models derived from intermediate-risk disease with either 10 μM ABT-737 (a BCL-2 inhibitor) as a positive control or 0–5 μM NER. At 48 hours after treatment, we dissociated the organoids, stained the cells with 7-aminoactinomycin D and conjugated antibodies against Annexin V, and quantified staining using flow cytometry. After gating each organoid model for live and dead cells following ABT-737 treatment (Figure 8A), we applied these gates to the NER-treated models (Supplemental Figure 7).

As summarized in Figure 8B, 2 models (183 and 187) demonstrated exquisite sensitivity to NER, with greater than 80% of cells dead at 3 μM and greater than 95% cells dead at 5 μM concentrations. By contrast, model 190 was mostly resistant, with less than 35% apoptotic at the 5 μM concentration. Intriguingly, model 188 displayed a mixed phenotype with approximately 50% and 65% of cells apoptotic at the 3 μM and 5 μM concentrations, respectively. Indeed, by multiplex immunofluorescence, P187 had a greater number of HER2-high and PSA/AR–low foci, whereas P190 harbored more HER2-low and PSA/AR–high foci (Figure 8C). In 3 of 4 cases, the histology and HALO AI cell segmentation accurately predicted pharmacologic behavior (Figure 8D). This range of responses, along with the bimodal distributions of cells observed by multiplex IF (Figure 8D), confirms that as the proportion of HER2-dependent cells changes between individuals, those preexisting subpopulations confer differential sensitivity to HER2 inhibition.

Finally, we reasoned that if preexisting and comingled cell populations were to be treated using an approach targeting both the AR activity–high and HER2 activity–high tumor cells, the exposure paradigm would need to be additive rather than synergistic, because the target cells are separate populations, as suggested by our organoid experiments (see Supplemental Figure 7). Using PCa cell lines to enable multiple independent replicates, we observed that a combination approach of enzalutamide and AFA in LNCaP cells at the drugs’ respective IC_20_ marginally decreased cell viability greater than the IC_20_ dose of enzalutamide or AFA alone (Figure 8E). By contrast, treatment with enzalutamide, AFA, or NER at an IC_50_ dose consistently killed approximately 50% of LNCaP cells, and the addition of AFA or NER to enzalutamide at its IC_50_ further improved cell killing to approximately 80% (*P*_adj_ < 0.0001, repeated measures ANOVA) and 65% (*P*_adj_ = 0.0004), respectively (Figure 8F). Similarly, VCaP cells treated with enzalutamide plus AFA or NER at their respective IC_50_ values improved cell killing to 97% (*P*_adj_ < 0.0001, repeated measures ANOVA) and 86% (*P*_adj_ = 0.0029), respectively (Figure 8G). Collectively, these data indicate that the improved antitumor effect of combining antiandrogen with HER2 inhibition is due to the simultaneous targeting of 2 distinct cell subpopulations.

Together, our findings indicate that localized PCas may be variably sensitive to HER2 inhibition or to combination AR and HER2 inhibition, depending upon the proportion of HER2-high/AR activity–low tumor cells contained therein.

## Discussion

Neoadjuvant therapies for PCa face inherent challenges of patient selection and the uncertainties of how subsequent therapies, upon recurrence, contribute to metastasis development. In this study, we identified patterns of baseline gene expression signatures that tracked with the volume of posttreatment residual disease, a surrogate pathologic biomarker for BCR (20). Our identification of HER2 activity association with poor responses to intense androgen deprivation neoadjuvant therapy is, in turn, associated with these tumors’ lower dependence on AR and thus decreased sensitivity to AR antagonism. We also demonstrated that HER2 inhibition, alone or in conjunction with AR-targeted therapies, has potent antitumor activity in the hormone-sensitive setting.

Notably, our present study demonstrates that HER2 expression in primary PCa represents a therapeutic vulnerability. HER2 has long been a tantalizing target in PCa, although most studies have been conducted in the metastatic and castration-resistant settings after tumors have recurred after androgen ablation. These prior studies commonly feature AR and HER2 in a single oncogenic axis (32, 34–37, 49, 50), with a notable exception describing opposing activities of AR and KLF5 centering on *ERBB2* expression (51). Collectively, the results presented in these prior studies (i.e., molecular mechanisms promoting AR activity in HER2-high tumor cells) reflect years of prolonged ADT for recurrent PCa, such that the therapeutic window for cotargeting HER2 and AR would have been lost. Indeed, the *AR* gene is amplified in 50%–80% of ADT-treated metastatic tumors, which results in marked reprogramming of cistromic and *cis*-regulatory elements governing gene expression (52, 53). In the hormone-sensitive setting, HER2 monotherapies have also been largely ineffective (49, 54), which may be explained in part by our finding that the proportion of AR- and HER2-dependent cells varies by patient, necessitating cotargeting. Our finding that approximately 60% of patients with high-risk localized PCa in the present study harbored moderate to high levels of HER2 protein expression in the absence of genomic amplification mirrors the findings of prior histologic analyses (55–57).

Although AR-targeted therapies alone are highly effective in the hormone-sensitive localized and metastatic settings, the addition of chemotherapy to ADT plus a novel hormonal agent has demonstrated that up-front intensification delays progression to castration resistance (58, 59). In the present study, we demonstrated sensitivity to HER2 monotherapy in ex vivo, patient-derived organoid models and HER2/AR combined inhibition using cell lines in vitro. Further confirmatory studies using in vivo preclinical models are necessary. With genomically informed neoadjuvant studies (e.g., ClinicalTrials.gov identifier NCT04812366) testing similar combinations in the presurgical setting, the work we report here nonetheless substantiates clinical investigation into the targeting of HER2 with or without intensive ADT, based in part on AR-high versus -low transcriptional activity or the proportion of each cell subset as determined by IHC. Indeed, this proportion may serve as a molecular determinant of ADT sensitivity, given that the AR activity–low cells are a preexisting subpopulation necessitating combination therapy at doses indicative of 2 independent targets (see Figures 7 and 8). Thus, because this subset of never-before-treated, de novo prostate tumors were showing little (or no) AR activity by transcriptional and histologic profiling, ADT would not be appropriate as a universal first-line systemic therapy.

In this study, we used transcriptional signatures of baseline HER2 activity based on the discovery of their robust correlation with posttreatment residual tumor volume. Canonically, HER2 activity is mediated downstream of ligands (e.g., EGF and NRG-1) resulting in phosphorylation of HER2 on tyrosine residues that accompany homodimerization and heterodimerization with EGFR and HER3 (60). Notably, these signatures correlated best with the steady-state cytosolic, but not membranous, levels of total HER2 protein detected by IHC in human tissues, with pHER2 detected at very low levels. Although this might reflect the challenges associated with IHC detection of phosphorylated epitopes in archival tissues, *ERBB2* is not amplified or mutated in any of these prostate tissues, and thus the pathologic grading standards applied to HER2^+^ lung and breast tumors may not necessarily apply. Although AR binds to the *ERBB2* locus, future investigation to assess the role of ligand-stimulated HER2 signaling in prostate tumor tissues will more clearly define the biological basis for HER2 activation beyond *ERBB2* expression and its role in promoting prostate tumor cell growth and survival in an AR activity–low state.

A distinction, as well as a limitation, of the present study was our use of prostate biopsy tissue from a neoadjuvant therapy clinical trial as a platform for discovery. Such abundance of biopsy tissue, made possible by the requirement of our treatment protocol for on-study targeted biopsies, was not possible for other neoadjuvant studies with molecular components recently published (10, 11, 24, 25). However, without well-defined transcriptomes across a broad range of tumor pathologic responses (i.e., RCBs), direct validation of the results of this study was not possible in a statistically meaningful secondary cohort. However, by using AR transcriptional activity as a surrogate marker of RCB, we found that AR activity and HER2 activity remain as distinctly inverse properties of prostate tumors that could be extended to other case sets. This inverse relationship of AR and HER2 activity, which, to our knowledge, has never been previously described in localized PCa, is similar to that recently reported for salivary gland cancers (61, 62). Salivary duct carcinoma normally expresses the AR, but a subset was shown to have higher levels of HER2 that corresponded to low or absent AR expression (62). Consistent with the present study’s findings, patients with high HER2/low-AR salivary duct carcinoma had worse prognoses (62).

Determining long-term disease-free survival benefits requires more extensive follow-up from the results of neoadjuvant therapy clinical trials, especially when conducted as platform studies with pathologic readouts. However, as we have shown here, a feature of prostate tumors to resist direct AR antagonism by enzalutamide is their intrinsic phenotype of having lower levels of AR activity. Therefore, even in the absence of extended clinical follow-up data, we have identified what we believe are previously unappreciated features of prostate tumor cells with direct clinical significance in their sensitivity to HER2 inhibition while demonstrating resistance to enzalutamide. Going beyond the population of patients with high-risk localized PCa, we propose that the AR activity–low population of metastatic hormone-sensitive PCa that would otherwise show poor response to enzalutamide may be targeted by HER2 inhibition, a hypothesis that could be readily tested in a genomically guided adjuvant therapy clinical study.

## Methods

### Sex as a biological variable.

Our study used specimens and cell lines derived only from male patients because PCa is only relevant in men.

### Biopsy specimen and radical prostatectomy selection.

Biopsy specimens for LCM and IHC were previously described (22). Briefly, patients who enrolled in a clinical trial of neoadjuvant ADT plus enzalutamide for 6 months underwent templated and magnetic resonance/ultrasound-fusion targeted biopsies prior to the initiation of therapy. Biopsy specimens were selected for LCM and IHC on the basis of tumor content and adverse pathologic features. Radical prostatectomy specimens from each patient were used to derive RCB volumes as a measure of absolute pathologic response, as previously described (8, 22, 63). Briefly, RCB was calculated by multiplying the slope of the regression line (0.4) by block thickness (0.6 cm), by the number of slices containing residual tumor, by the largest cross-sectional width and length of that tissue, and by tumor cellularity. We considered patients with an RCB < 0.05 cm^3^ as exceptional responders and we grouped INR patients into a single category for RCB > 0.05 cm^3^. Residual tumor was identified using a combination of routine H&E and additional immunostains to verify the presence of residual tumor, including anti-NKX 3.1, PIN-4 cocktail, CAM5.2, and anti-p63 as previously described (8, 22).

All prostatectomy blocks harboring residual tumor were used for immunostains against EGFR, pEGFR, HER2, pHER2, HER3, and pHER3. One biopsy block per case was used for immunostains against HER2. The numbers of cases with biopsy blocks harboring sufficient remaining tumor material for additional IHC were as follows: anti-EGFR, 35; anti-pHER2, 31; and anti-pEGFR, 29.

A separate cohort comprising radical prostatectomy specimens from 61 patients (described in ref. 64) who received no prior therapy was used for bulk RNA-Seq and multiplex staining of HER2, AR, and PSA. Each block containing tumor tissue was selected from the index lesion of each tumor.

Organoids derived from patients with PCa were acquired from fresh prostatectomy tissue in patients undergoing radical retropubic surgery at Emory St. Joseph Hospital (Atlanta, GA). Tumor tissue was identified jointly by the attending surgeon and pathologist at the time of tissue grossing.

Frozen prostatectomy specimens (embedded in OCT) from the Prostate Cancer Biorepository Network (PCBN) were sectioned onto glass slides and stained with H&E, or were shipped as ribbon curls in microfuge tubes. After review by a genitourinary pathologist (RTL) to confirm the presence of tumor cells, ribbon curls were processed using the RNeasy Plus Mini Kit (Qiagen) to extract total RNA.

### Tissue processing.

Diagnostic H&E slides of FFPE biopsy tissue were reviewed by 2 genitourinary pathologists (RTL and HY). Additional immunostains for tissue characterization and/or to guide LCM were performed on 5 μm serial sections of tissue, as previously described (22). All IHC assays were performed using validated protocols on a PATH FLX autostainer (Biocare Medical). For LCM, additional 5 μm serial sections of tissue were cut onto polyethylene naphthalate membrane slides (MicroDissect), with glass slides cut after every 5 membrane slides for additional H&E and IHC stains to serve as references. Membrane slides were baked, deparaffinized, rehydrated, and stained with Paradise stain (ThermoFisher). Approximately 10,000–50,000 tumor cells per ROI were captured from serial sections, using an ArcturusXT Ti microscope onto CapSure Macro LCM Caps (ThermoFisher) using the infrared capture and UV cutting lasers. Adjacent stromal tissue that was incidentally captured was ablated using the UV laser. Micrographs of each cap were taken after each LCM session and cross-referenced against reference slides by 2 blinded genitourinary pathologists (RTL and HY) to verify the regions captured. RNA was extracted from LCM tissues by using a clean scalpel to excise the LCM cap polymer and immerse it in Buffer PKD from the RNeasy FFPE Mini Kit (Qiagen). Full laboratory procedures for tissue staining can be found in the Supplemental Data.

### Cell culture and protein analysis.

LNCaP, 22Rv1, and VCaP cells were purchased from the American Type Culture Collection. LAPC-4 cells were a gift from Charles Sawyers, Memorial Sloan Kettering, New York, NY, USA. Cells were authenticated by STR profiling (Laragen) every 6 months. LNCaP and 22Rv1 cells were maintained in RPMI-1640 containing 10% FBS, 1% penicillin-streptomycin solution, and 1% l-glutamine. LAPC-4 cells were maintained in IMDM containing 10% FBS, 1% penicillin-streptomycin solution, and 1% l-glutamine. VCaP cells were maintained in DMEM containing 10% FBS, 1% penicillin-streptomycin solution, and 1% l-glutamine.

Dose-response curves were performed with 22Rv1, LAPC-4, LNCaP, and VCaP cells for each new lot of drug acquired. Abiraterone, enzalutamide, AFA, ERL, DAC, GEF, LAP, NER, SUN, and VAN were acquired from the National Cancer Institute Developmental Therapeutics Program Open Chemicals Repository Collection. For each assay, cells were seeded in solid, white, flat-bottom, tissue culture–treated 96-well plates, at 5,000 cells per well in a volume of 100 μL. Blank wells were filled with 150 μL of complete medium. Unused wells were filled with 75 μL of PBS. Each agent was prepared in DMSO (vehicle) in 50 μL at 3 times its intended concentration for a final volume per well of 150 μL. Drug concentrations were tested at 100, 50, 10, 5, 1, 0.1, and 0 μM 1 day after plating, and cell viability was measured after 5 days of treatment using CTG 2.0 Cell Viability Assay Reagent (Promega; G9243). Prior to measurement, plates were left to equilibrate for 30 minutes at room temperature (RT), 75 μL of CTG reagent was added per well, and plates were read using a Tecan Infinite M200 Pro with 1.5 mm of orbital shaking for 2 minutes, 10 minutes of incubation, and luminescence reading with integration time for 1,000 milliseconds.

Cells were treated with each agent at its IC_50_ (or IC_20_), alone or in combination, to measure tumor cell viability, RNA levels, or protein levels. For drug treatments to assess viability, cells were seeded the same way, treated 1 day after plating, and grown for 5 days prior to incubation with CTG. Viability measurements were performed at least 5 times independently. For RNA and protein extraction, cells were seeded in 6-well plates at 50,000 cells per well in a volume of 2 mL. Cells were grown to 75% confluency prior to drug treatment. Cells were grown for 5 days, treated for 5, 3, 1, or 0 days, in duplicate for RNA and protein extraction, at least 3 times independently.

RNA was extracted using the RNeasy Plus Mini Kit, scraping cells directly into Buffer RLT Plus. Protein was extracted by scraping cells into RIPA buffer (Pierce; 89900) supplemented with Halt Protease and Phosphatase Inhibitor Cocktail (ThermoFisher; 78440). Lysates were incubated on ice for 10 minutes, vortexed, and centrifuged at 21,000*g* for 10 minutes at 4°C. Supernatants were stored at –80°C.

Protein lysates were separated by SDS-PAGE on 4%–15% Criterion TGX protein polyacrylamide gels (Bio-Rad) and transferred to nitrocellulose membranes via semi-dry transfer. After 1 hour of blocking in 10% nonfat dry milk in TBST, membranes were incubated with the following primary antibodies overnight at 4°C, all diluted into TBST with 5% BSA: anti-AR clone D6F11 (Cell Signaling; 5153), anti-PSA clone D6B1 (Cell Signaling; 5365), and anti-actin clone C4 (Millipore; MAB1501). Membranes were washed and incubated with HRP-conjugated secondary antibodies (1:5,000–10,000 dilutions) for 1 hour, reacted with Clarity Western ECL substrate (Bio-Rad; 1705061) and visualized using the ChemiDoc Touch Imaging (Bio-Rad) system.

For flow cytometry against HER2, LNCaP cells were plated in 10 cm dishes at a density of 400,000 cells per plate. Cells were grown overnight and treated with enzalutamide for 0, 24, or 72 hours. Cells were rinsed once with cold PBS and harvested by scraping into microfuge tubes, using cold FACS Buffer (PBS containing 2% FBS with 1% 0.5 M pH 8.0 EDTA). Cells were subjected to viability staining with the LIVE/DEAD Fixable Aqua Dead Cell Stain kit for 405 nm excitation (ThermoFisher; L34966) for 1 hour on ice. Cells were fixed using 4% paraformaldehyde for 5 minutes at RT and permeabilized with 0.5% Tween-20 for 5 minutes at RT. After washing, cells were stained with HER2 antibodies conjugated to APC/Fire 750 (24D2) (1:50 dilution; BioLegend, 324422) for 30 minutes at RT. Cells were subjected to Vector TrueVIEW Autofluorescence Quenching Kit (Vector; SP-8400-18) at a 1:1:1 ratio for 5 minutes at RT. Samples were acquired on a BD FACSymphony A5 cell analyzer and analyzed using FlowJo 10. Each treatment group was compared with the day 0 control. MFI was calculated from the global population for all treatment groups, and the top 20% HER2 expression was calculated as all fluorescence intensity above the 20th percentile for each biological replicate.

### Patient-derived organoids.

Fresh tumor tissues from prostatectomies were minced and digested in basal medium (Advanced DMEM/F12 containing Glutamax, HEPES, and antibiotics) containing 5 mg/mL collagenase type II, and 10 μM Rho-kinase inhibitor (Y-27632) overnight at 37°C. Erythrocytes were lysed in 1 mL of RBC lysis buffer for 5 minutes at RT, followed by centrifugation at 300*g*. The cell pellet was resuspended in growth factor–reduced Matrigel (Corning; 354230) and seeded as ~20,000 cells in a 40 μL drop in the middle of a 24-well plate. Prostate organoids were cultured in basal medium containing the following: 50× B-27, 10% R-spondin–conditioned medium, 5 ng/mL EGF, 10 ng/mL FGF-10, 5 ng/mL FGF-2, 1.25 mM *N*-acetylcysteine, 10 μM Y-27632, 500 nM A-8301, 10 mM nicotinamide, 100 ng/mL Noggin, 1 μM prostaglandin E2, 10 μM SB202190, and 1 nM dihydrotestosterone. Medium was changed every 2–3 days. Organoids appear within 7 days after plating and passaging at a 1:4 dilution every 1–3 weeks with TrypLE Express containing 10 μM Y-27632, followed by mechanical dissociation to single cells.

Organoids were plated at a density of 20,000–30,000 cells per well and treated with ABT-737 (MedChemExpress; HY-50907) (10 μM) or NER at 1, 3, and 5 μM for 72 hours. Cells were harvested, spun down, and washed twice with 1× cold PBS. Cells were subjected to viability staining with the Pacific Blue Annexin V Apoptosis Detection Kit with 7-aminoactinomycin D (BioLegend; 640926). Samples were acquired on a BD FACSymphony A3 cell analyzer and analyzed using FlowJo 10.

### Library preparation and sequencing gene expression profiling.

Up to 100 ng of RNA extracted from FFPE tissues (Dana-Farber/Harvard Cancer Center cohort), up to 1 μg of RNA extracted from frozen tissue sections (Prostate Cancer Biorepository Network cohort), and 1 μg of RNA from cell lines were used for preparing paired-end Illumina-compatible RNA-Seq libraries. Tissue-derived RNA was first processed using the NEBNext Globin & rRNA Depletion Kit (New England Biolabs), while cell line–derived RNA was processed using polyA capture. Enriched RNA was assembled using the NEBNext Ultra II Directional RNA Library Prep Kit and sequenced on an Illumina NovaSeq system. Full procedures for bioinformatic processing can be found in the Supplemental Data.

### Library preparation and sequencing for ChIP-Seq.

Approximately 10 million LNCaP cells were fixed using 2 mM DSG (CovaChem) for 10 minutes, followed by 1% formaldehyde for 10 minutes at 37°C, and quenched with 2 M glycine for 5 minutes at RT. Cross-linked cells were resuspended in cold lysis buffer (50 mM Tris pH 8.0, 10 mM EDTA, 0.5% SDS, 1× protease inhibitor cocktail) and sheared using a Bioruptor Pico device (Diagenode). Fragmented chromatin was incubated with 3 μg of AR antibody (Abcam; 108341) overnight at 4°C. Input was 5% of each sample prior to addition of antibody. Protein A/G beads (ThermoFisher) were added and incubated for 1 hour at 4°C, washed 6 times with RIPA buffer (50 mM HEPES pH 7.5, 1 mM EDTA pH 8.0, 500 mM LiCl, 0.7% sodium deoxycholate, 1% NP40), and eluted in 100 mM NaHCO_3_, 1% SDS. Samples, including input DNA, were treated with RNase A (ThermoFisher) for 30 minutes at 37°C followed by proteinase K (New England Biolabs) at 65°C overnight to reverse crosslinking. ChIP DNA was purified with Monarch Genomic DNA Purification Kit (New England Biolabs) and concentrations were quantified by Qubit (ThermoFisher) and TapeStation (Agilent). Libraries were prepared using the NEBNext Ultra II DNA Library Prep Kit (New England Biolabs) and sequenced on the Illumina NextSeq platform. Full procedures for bioinformatic processing can be found in the Supplemental Data.

### Statistics.

Statistical analyses were performed using GraphPad Prism 10, Microsoft Excel for Mac 16, and R 4.2.0. Comparisons of genes based on RCB were performed using linear mixed-effects models with up to 2 fixed effects. Pathway enrichment was filtered and sorted by *P* values, adjusted *P* values, and bias-corrected *z* scores. Associations between factors were measured using Pearson and Spearman correlations and log-ratio tests. Comparisons of cell abundance were performed using ANOVA tests with Bonferroni adjustment for multiple comparisons or with Friedman tests with Dunn’s post hoc test.

### Study approval.

All specimens were used in accordance with the terms of informed written consent provided by study participants and patients, in accordance with the principles of the Declaration of Helsinki. The collection and analysis of tissue from patients with localized PCa treated by neoadjuvant ADT plus enzalutamide were approved by the NIH IRB (protocol 15-c-0124; ClinicalTrials.gov identifier NCT02430480). Tissue samples were acquired from the PCBN through an agreement with the University of Washington Genitourinary Cancer Specimen Biorepository (agreement 888). Organoids derived from patients with PCa were acquired and grown at Emory University under an Emory IRB–approved protocol (STUDY00005649). Standard-of-care prostatectomy specimens were used in accordance with protocols 15-008 and 15-492 from the Dana-Farber/Harvard Cancer Center.

### Data availability.

The data underlying this article have been deposited in the Database of Genotypes and Phenotypes (https://www.ncbi.nlm.nih.gov/gap/) (phs001938.v3.p1 and phs001813.v3.p1) and the Gene Expression Omnibus (GSE183019, GSE183100, GSE201284, GSE183126, GSE152516, GSE222196, GSE302203, and GSE304127) (https://www.ncbi.nlm.nih.gov/geo/). Underlying data values are presented in the Supporting data values file.

## Author contributions

SW, IMK, DL, SYT, JRB, NTT, AB, KEH, CS, BV, NCW, NVC, RA, and RL contributed to data acquisition. SW, ATK, SYT, JRB, AB, ERS, JMF, and CSJ contributed to methodology. HTK, PAP, PLC, BT, WLD, and FK provided reagents and/or patients. SW, ATK, SK, RTL, NTT, AB, CL, HY, and SAH contributed to data analysis. SW, DYT, HTK, and AGS supervised the study. All authors contributed to drafting, reviewing, and editing the manuscript. Authorship order among the co-first authors was determined by chronological order in which they began work on this project.

## Funding support

This research was supported in part by the Intramural Research Program of the NIH and is subject to the NIH Public Access Policy. Through acceptance of this federal funding, the NIH has been given a right to make the work publicly available in PubMed Central. The contributions of the NIH authors are considered works of the U.S. government. The findings and conclusions presented in this article are those of the authors and do not necessarily reflect the views of the NIH or the U.S. Department of Health and Human Services. Portions of this work used the computational resources of the NIH HPC Biowulf cluster.

- Prostate Cancer Foundation (Young Investigator Awards to SW, ATK, and SAH).
- Department of Defense Congressionally Directed Medical Research Program, Prostate Cancer Research Program (PCRP) Early Investigator Awards W81XWH-19-1-0712 (to SW) and W81XWH-22-1-0067 (to ATK).
- PCRP Impact Award W81XWH-16-1-0433 (to AGS).
- PCRP Prostate Cancer Biorepository Network (W81XWH-18-2-0013, W81XWH-18-2-0015, W81XWH-18-2-0016, W81XWH-18-2-0017, W81XWH-18-2-0018, and W81XWH-18-2-0019).
- NIH Medical Scientist Training Program Grant T32GM008169 (to CSJ).
- NIH Predoctoral Award F30CA243250 (to CSJ).
- Intramural Research Program of the National Cancer Institute.

## Supplementary Material

Supplemental data

ICMJE disclosure forms

Unedited blot and gel images

Supporting data values

## Version 1 09/04/2025

In-Press Preview

## Version 2 11/17/2025

Electronic publication
