# Supplementary material for: Localized high-risk prostate cancer harbors an androgen receptor activity–low subpopulation susceptible to HER2 inhibition
Source: J Clin Invest. 2025 Sep 4;135(22):e189900. doi: 10.1172/JCI189900 (PMC12618079; doi:10.1172/JCI189900)
Supplement: Supplemental data [file jci-135-189900-s311.pdf]

Supplemental Information

Localized high-risk prostate cancer harbors an androgen receptor-activity low subpopulation susceptible to HER2 inhibition

by

Scott Wilkinson and Anson T. Ku et al.

Supplemental Methods
Supplemental Figures
Supplemental References

SUPPLEMENTAL METHODS

Cell culture and protein analysis

For growth factor stimulation, LNCaP cells were washed twice with PBS the day after plating and media was replaced with growth media supplemented with 10% charcoal/dextran treated FBS (CSS, HyClone; SH30063.03). The next day, cells were treated with either 100 ng/ml EGF (R&D; 236-EG) or 100 ng/ml NRG-1 (R&D; 396-HB) for 5 m, 1 h, or 4 h. RNA was extracted using the RNeasy Plus Mini Kit (Qiagen) following the manufacturer's protocol, scraping cells directly into 2× loading buffer (Bio-Rad; 161-0737). Protein was extracted by scraping cells into RIPA buffer (Pierce; 89900) supplemented with Halt Protease and Phosphatase Inhibitor Cocktail (Thermo Scientific; 78440). Lysates were incubated on ice for 10 m, vortexed, and centrifuged at 21,000 × g for 10 minutes at 4°C. Supernatants were sonicated briefly and stored at -80°C.

Protein lysates were separated by SDS-PAGE on 4–15% Criterion TGX protein polyacrylamide gels (Bio-Rad) and transferred to nitrocellulose membranes via wet transfer. After 1 h blocking in EveryBlot (Bio-Rad; 12010020), membranes were incubated with the following primary antibodies for 1 h, all diluted into EveryBlot: anti-HER2 clone 29D8 (Cell Signaling; 2165), anti-phospho-HER2 Y1221/1222 clone 6B12 (Cell Signaling; 2243) and GAPDH clone 6C5 (Millipore; MAB374). Membranes were washed and incubated with HRP-conjugated secondary antibodies (1:5000–10000 dilutions) for 1 h, reacted with Clarity Western ECL substrate (Bio-Rad; 1705061) and a Gel Doc (Azure Biosystems) system.

16-bit grayscale images were exported from the Gel Doc and imported into ImageJ (NIH). Equivalent size polygons were used for background correction of each band. Normalized pHER2 signal for each sample was based on the signal density of the pHER2 band, its corresponding total HER2 band, and GAPDH (as a loading control).

Bioinformatic processing

Gene expression and pathway analysis

FASTQ files were processed as previously described (1). Briefly, reads were trimmed using Trimmomatic version 0.36, and gene-level counts were estimated using RSEM version 1.3.2 as a wrapper around STAR version 2.7.0f in stranded mode. Gene fusions were identified using defuse version 0.8.1. FASTQ pairs of TCGA data were downloaded from the NCI Genomic Data Commons and processed using an identical pipeline. Raw counts were normalized using the trimmed mean method in edgeR package in R, and used to generate heatmaps with the pheatmap package.

Differentially-expressed genes per unit of residual cancer burden (RCB) were derived using the variancePartition (2) package, modeling patients as random effect, using the formula ~RCB + (1|Patient) with only RCB as the fixed effect, or ~RCB + ERG/PTEN (1|Patient) incorporating ERG or PTEN as additional fixed effects in the linear mixed-effects model. The β coefficient for the fixed effect was used as the magnitude of gene expression change. Alternatively, AR enrichment score for each sample was calculated using the gene set "Hallmark of androgen response" and with the GSVA package in ssgsea mode with tau = 0.75. Next, differentially-expressed genes (DEGs) associated with AR activity were derived using ~AR + (1|Patient) in variancePartition. For the TCGA and PCBN cohorts, DEGs were identified using linear regression models with the DESeq2 package. Prior exome sequencing (3) of the same laser capture microdissected exomes were manually curated using IGV to report the mutation status

and somatic copy number estimates for PTEN, RB1 and TP53.

Gene expression (v.21Q1), drug sensitivity (v.19Q4) and RNAi (Broad/Novartis/Marcotte) viability screen data were downloaded from the Broad Institute DepMap (depmap.org). Gene expression data was processed using the ssGSEA module of GenePattern (genepattern.org) to measure HER2 and AR activity gene sets from mSigDB.

LNCaP single-cell gene expression data was downloaded from the NIH Gene Expression Omnibus (GEO) using accession ID GSE168668. Data was processed using cellranger version 7.2.0 and aligned to GRCh38-2020-A. The resulting cells were filtered based on total gene counts, number of unique reads and mitochondrial fractions. Next, the data were normalized using computeSumFactors from the scran package and integrated using fastMNN from the batchelor package. Clustering was performed using Louvain method with clusterCells from the scran package. K was calculated using clusGap from the cluster package. Trajectory analysis was performed on the batch-corrected embedding using the slingshot package.

Differential gene analysis from treated prostate cancer cell lines over a time course was performed with formula "~ day" in voomWithWeights and dream from the variancePartition package. The final bias-corrected z scores were derived by averaging the two replicates from each treatment condition (cell line, drug and day).

To identify differentially-expressed genes of PCa tissues based on HER2/PSA ratio scores, samples with total counts greater than 10^6 were retained, and the median HER2/PSA ratio for each sample was used as the fixed effect with DESeq2 using the log-ratio test. Differentially-expressed genes were determined by adjusted P value of less than 0.1.

Pathway enrichment using the upstream regulator module were performed using Ingenuity Pathway Analysis (releases 2022 Q1 through 2025 Q2). Candidate upstream regulators were filtered for genes, pathways, and transcription factors and sorted by bias-adjusted z-score, with or without false discovery correction using the Benjamini-Hochberg method.

Breast cancer cell line gene expression data was downloaded from GEO using accession IDs GSE79776 and GSE237607. Breast cancer and LNCaP cells stimulated with EGF and/or NRG at different time points were matched with their controls and used to derive differentially-expressed genes using DESEQ2 with design formula set to "~id + batch". Samples with less than 1M counts were removed. Genes with less than 5 counts in 3 samples were filtered. DEG analyses were performed with batch correction using "id" as a factor of comparison (for each timepoint or growth factor). Genes with $\log_2$ fold-change greater than 1 or less than -1 and adjusted P value of less than 0.05 were retained and used as input into Ingenuity Pathway Analysis and as candidate genesets with single-sample GSEA.

TPM normalized data of laser capture microdissected tumor foci were used to compute single sample GSEA scores using the GSVA package in ssGSEA mode using the LNCaP NRG-1 or EGF stimulated DEGs as genesets.

Chromatin immunoprecipitation

FASTQ reads were aligned to build hg19 after filtering for ENCODE blacklist regions using bwa version 0.7.17 and converted to BAM format using Samtools version 1.17. Duplicate reads were removed using Picard version 2.27.3 and peaks identified with MACS2 version 2.2.7.1 using input controls with a

q- value cutoff of 0.01. Input-subtracted bigWig files were generated using Deeptools version 3.5.1 for visualization in IGV. Quality assessment included sequence quality scores, non-redundant fraction, fraction of reads falling within peak regions, overlap with known DNase I hypersensitivity sites derived from ENCODE, and motif enrichment by Homer.

H3K27ac ChIP data of LNCaP cells treated with 5 μM enzalutamide or DMSO for 14 d were downloaded from GEO using accession ID GSE215945. H3K27ac HiChIP data of LNCaP cells was downloaded from the WashU Epigenome Browser (https://wangftp.wustl.edu/~dli/Claudia/HiChIP_LNCaP.gz).

Immunohistochemistry and immunofluorescence

For IHC against EGFR, pEGFR, HER2, pHER2, HER3 and pHER3, glass slides containing tissue sections were baked for 30 minutes at 60°C. Following deparaffinization in xylenes and rehydration through graded alcohols, antigen retrieval was performed using a NxGen Decloaker (Biocare Medical) at 110°C for 15 minutes in Tris-EDTA Buffer (Abcam; ab93684), pH 9.0. Next, a thin border was drawn around the edges of each glass slide using a PAP pen. After 10-minute incubations in Background Punisher (Biocare; BP974), 300 μL of primary antibody solutions were prepared and incubated with tissues at room temperature at 1:100 dilutions into Renoir Red diluent (Biocare Medical; PD904) for 1 h: anti-EGFR clone D38B1 (Cell Signaling; 4267), anti-pEGFR clone D7A5 (Cell Signaling; 3777), anti-HER2 clone 29D8 (Cell Signaling; 2165), anti-pHER2 clone 6B12 (Cell Signaling; 2243), anti-HER3 clone D22C5 (Cell Signaling; 12708), and anti-pHER3 clone 21D3 (Cell Signaling; 4791). Secondary detection was achieved with Mach 4 (Biocare Medical; M4U534H) polymer and/or probe for 30 minutes. Chromogen development was achieved with Betazoid DAB (Biocare Medical; BDB2004) and counterstained with CAT hematoxylin (Biocare Medical; CATHE) diluted 1:3 into distilled water. Slides were dehydrated through graded alcohols into xylene, mounted using Permount (Thermo Fisher), and digitized using a Carl Zeiss AxioScan.Z1 microscope slide scanner equipped with a Plan-Apochromat 20× NA 0.8 objective.

For multiplex immunofluorescence of AR, PSA and HER2, slides underwent preprocessing, antigen retrieval, and blocking as described above. Anti-HER2 clone 29D8 (Cell Signaling; 2165) was diluted 1:100 in 1× Antibody Diluent/Block (Akoya Biosciences; ARD1001EA) before incubating for 1 h. Slides were washed with TBST then incubated with ImmPRESS HRP Goat-anti-Rabbit IgG Polymer Reagent (Vector Laboratories; 30125) for 1 h. Slides were washed with TBST then incubated with Opal 650 (Akoya Biosciences; OP-001005) diluted 1:150 in 1× Plus Amplification Diluent (Akoya Biosciences; FP1498) for 1h. The next day, slides were removed and washed in diH$_2$O. Antibodies were stripped using HIER with Diva Decloaker (Biocare Medical; DV2004MX) in a NxGen Decloaking Chamber at 110°C for 30 m. After cooling, slides were loaded onto the PATH FLX autostainer. Slides were washed with TBST, then blocked using Background Punisher for 1 h. AR clone D6F1T (Cell Signaling; 5153) was diluted 1:100 in 1× Antibody Diluent/Block (Akoya Biosciences; ARD1001EA) before incubating for 1 h. Slides were washed with TBST then incubated with ImmPRESS HRP Goat-anti-Rabbit IgG Polymer Reagent (Vector Laboratories; 30125) for 1 h. Slides were washed with TBST then incubated with Opal 520 (Akoya Biosciences; OP-001001) diluted 1:150 in 1× Plus Amplification Diluent (Akoya Biosciences; FP1498) for 1 h. The next day, slides were removed and washed in diH$_2$O. Antibodies were stripped using HIER with Diva Decloaker in a NxGen Decloaking Chamber at 110°C for 30 m. After cooling, slides were loaded onto the PATH FLX autostainer. Slides were washed with TBST, then blocked again using Background Punisher for 1 h. PSA clone D6B1 (Cell Signaling; 5365) was diluted 1:50 in Renoir Red Diluent and incubated with tissues for 1 h. After washing with TBST, goat anti-Rabbit IgG (H+L) Secondary Antibody, AlexaFluor 555 (ThermoFisher Scientific; A-21428) was diluted 1:50 in Renoir Red

Diluent and incubated for 1 h. Slides were washed with TBST, then removed from the autostainer. Slides were washed with diH$_2$O, then incubated with Vector TrueVIEW Autofluorescence Quenching Kit (Vector Laboratories; SP-8400) for 2 m. Slides were washed with diH$_2$O, then incubated with 350 nM DAPI (4',6-Diamidino-2-Phenylindole, Dihydrochloride) (ThermoFisher Scientific; D1306) for 10 m. Slides were washed with diH$_2$O then mounted using ProLong Glass Antifade Mountant (Invitrogen; P36980). As controls for efficient and complete antibody stripping, additional control slides were processed with each one of the primary antibodies described, completing all other steps but omitting the other two primary antibodies. Slides were then digitized on Carl Zeiss AxioScan.Z1 microscope slide scanner equipped with a Plan-Apochromat 20× NA 0.8 objective equipped with a Colibri 7 flexible light source, and post-processed using ZenBlue (Zeiss).

Fully quantitative IHC analyses against AR, PSA, SYP, GR and Ki-67 were generated using Definiens software and reported previously (3). Semi-quantitative IHC analyses against EGFR, p-EGFR, HER2, p-HER2, HER3 and p-HER3 were performed using the H-scoring approach, which considers the proportion of tumor cells per slide that display no (0), low (1), medium (2) or high (3) staining intensity. The proportion of cells is multiplied by the intensity factor, for a maximum possible score per slide of 300 (100% of cells at score 3). H-scoring was conducted separately for cytosolic and membranous staining with distinct scores recorded for invasive vs. intraductal tumor histologies. The maximum H-score for each case was used for further comparisons.

Fully quantitative multiplex IF analyses against AR, HER2 and PSA were performed using HALO AI version 3.6 (Indica Labs) with the DenseNet V2 network and HiPlex-FL analysis routine for HALO AI. Each slide was individually trained and classified with at least 1000 iterations to achieve per-slide entropies of < 0.01 for tumor detection. Fluorescent detection settings for AR, HER2 and PSA were identical for all slides and applied to the tumor mask only. To calculate the HER2/PSA ratio scores, fully-segmented cells containing values for nuclei, cytoplasm and membrane were first filtered by cell size to include only objects with cell areas 10–120 μm^2 in size, HER2 membrane completeness greater than 0, and then determining a ratio defined by [((HER2 membrane intensity × HER2 membrane completeness) ÷ PSA cell intensity) + AR nuclear intensity].

SUPPLEMENTAL FIGURES

Supplemental Figure 1

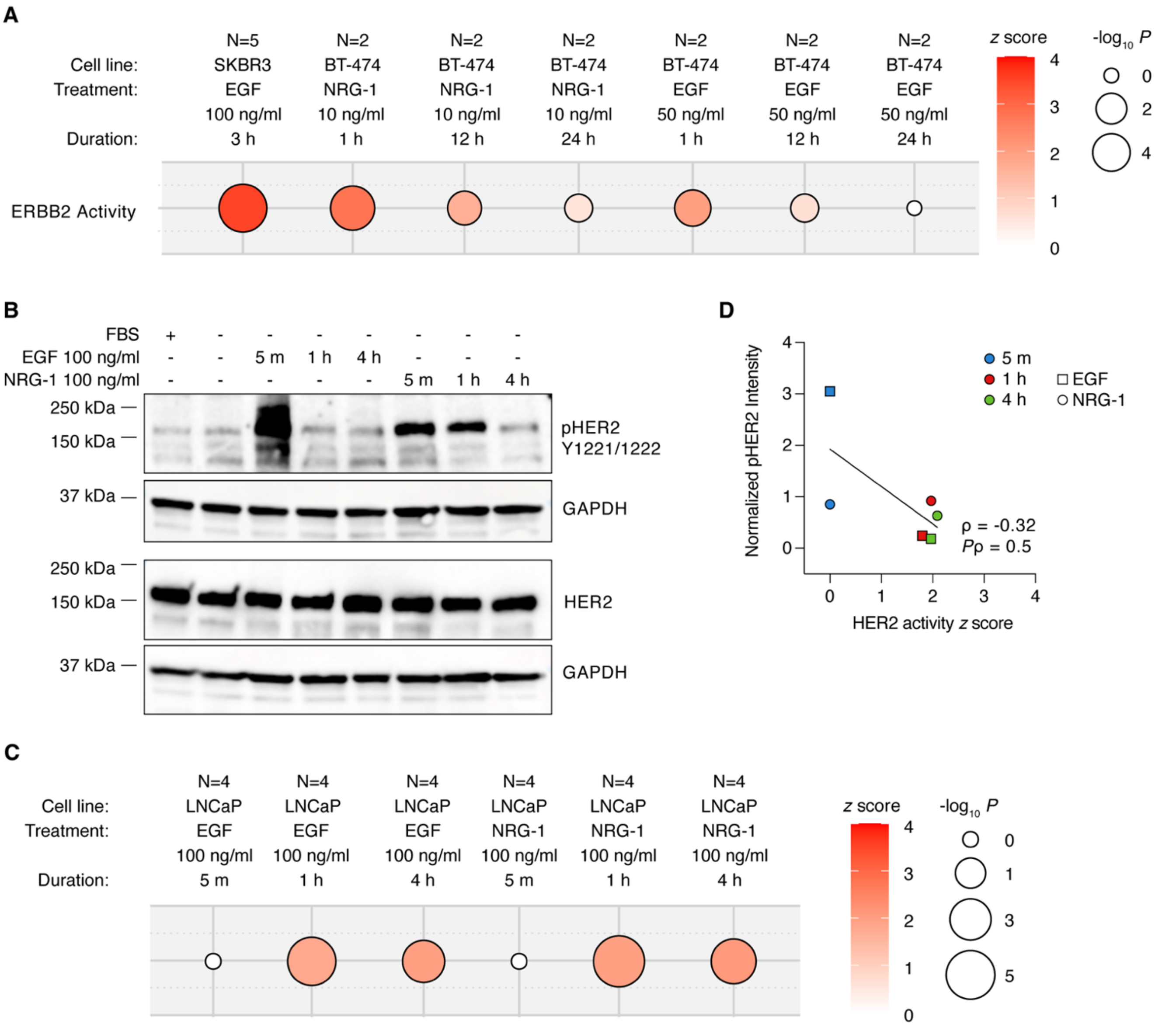

Supplemental Figure 1. Analytical validation of *ERBB2* Upstream Regulator signature. (A) *Differentially-expressed genes were derived from HER2+ (ERBB2-overexpressing) breast cancer cell line datasets (GSE79776 and GSE23760) in which SKBR3 or BT-474 cells were treated with growth factors (vs. controls) and processed with the upstream regulator module of Ingenuity Pathway Analysis. The number of unique replicates, ERBB2 activation z score and P value are shown.* **(B–D)** *LNCaP cells serum-starved overnight and treated with growth factors. Protein* **(B)** *or RNA* **(C–D)** *were extracted from cells at the given timepoints.* **(B)** *Immunoblot against pHER2 and HER2 is representative of four independent experiments.* **(C)** *Differentially-expressed genes were derived growth factor-stimulated cells vs. controls and processed with the upstream regulator module of Ingenuity Pathway Analysis. The number of unique replicates, ERBB2 activation z score and P value are shown.* **(D)** *Densitometry against pHER2 was performed relative to total HER2 and GAPDH. Scatter plot depicts average (N=4) normalized pHER2 intensity (y-axis) depicted in* **(B)** *vs. HER2 activity z score (x-axis) depicted in* **(C)**.

Supplemental Figure 2

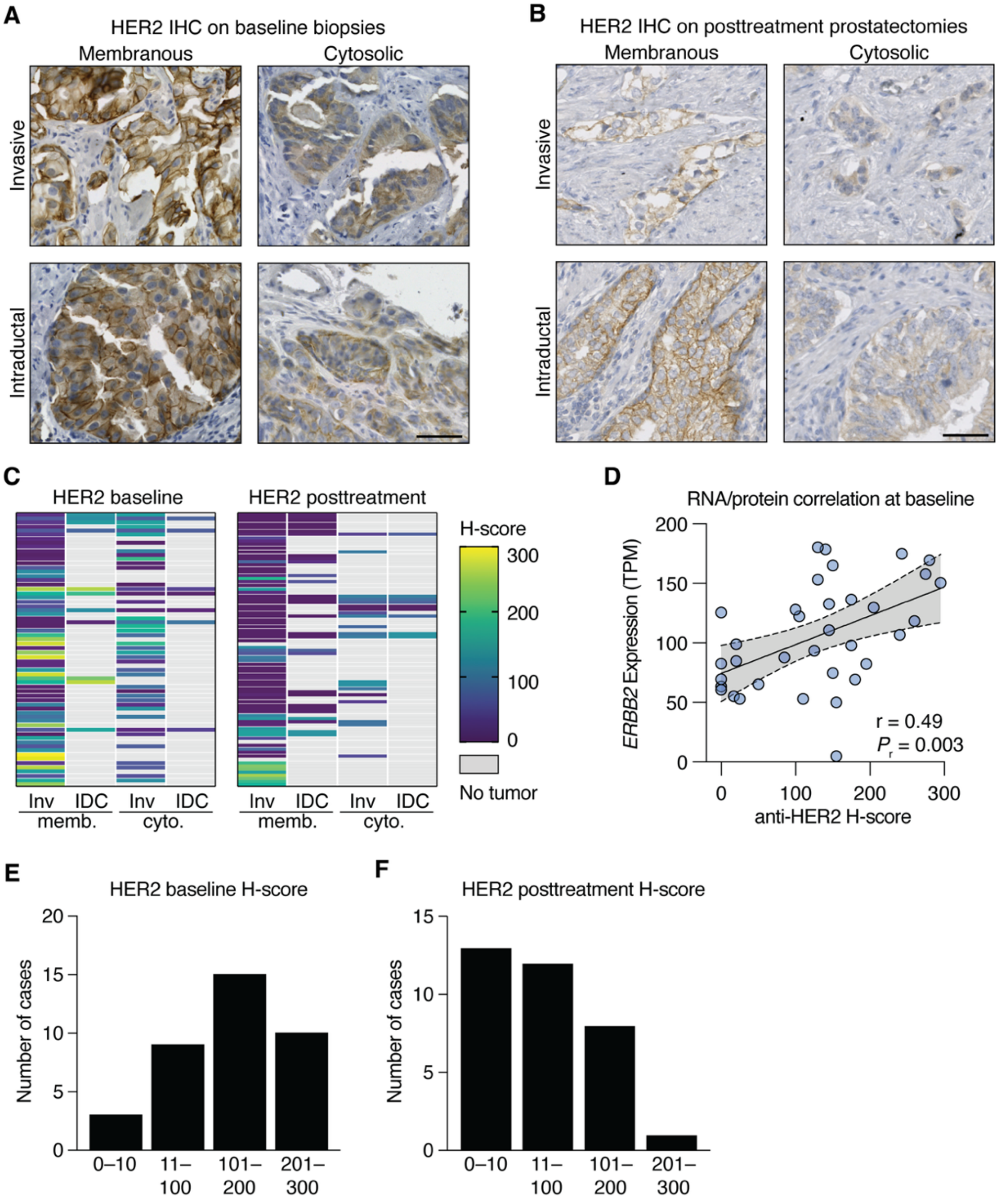

Supplemental Figure 2. Immunohistochemistry against HER2. *Examples are shown of invasive and intraductal morphologies with membranous and cytosolic staining in baseline biopsy* **(A)** *and posttreatment radical prostatectomy* **(B)** *specimens. Bar: 50 µm. Inv: invasive; IDC: intraductal carcinoma; memb: membranous; cyto: cytosolic.* **(C)** *Heatmaps summarizing semi-quantitative analysis of anti-HER2 immunohistochemistry (IHC) performed on entire sections of biopsies (n = 69) and posttreatment surgical specimens (n = 81). Rows are grouped by patient.* **(D)** *Scatter plot showing the association of matched foci (n = 35) of HER2 baseline H-scores (x-axis) with gene expression of ERBB2 transcripts per million (TPM) (y-axis). Statistical significance determined using Pearson's correlation.* **(E–F)** *Density plots summarizing the frequency distribution of anti-HER2 IHC semi-quantitative analysis, per patient, of baseline biopsies* **(E)** *and radical prostatectomies* **(F)**.

Supplemental Figure 3

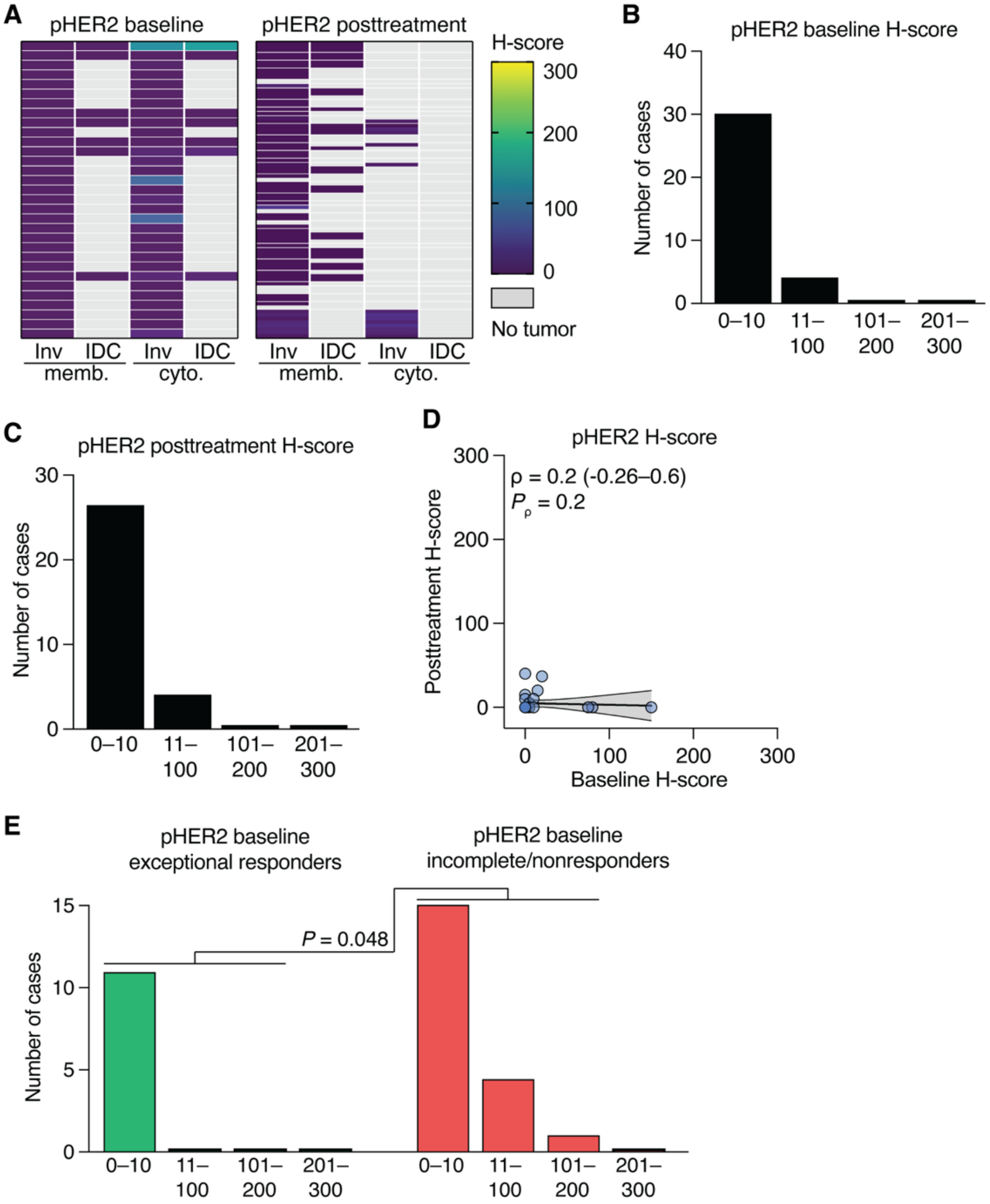

Supplemental Figure 3. Immunohistochemistry against pHER2. (A) *Heatmaps summarizing semi-quantitative analysis of anti-pHER2 immunohistochemistry (IHC) performed on entire sections of biopsies (n = 37) and posttreatment surgical specimens (n = 79). Rows are grouped by patient. Inv: invasive; IDC: intraductal carcinoma; memb: membranous; cyto: cytosolic.* **(B–C)** *Density plots summarizing the frequency distribution of anti-pHER2 IHC semi-quantitative analysis, per patient, of baseline biopsies* **(B)** *and radical prostatectomies* **(C)**. **(D)** *Scatter plot showing the association of pHER2 baseline H-scores (x-axis) with posttreatment H-scores (y-axis). Statistical significance determined using Spearman's rank correlation. Line and gray shaded area show the linear regression line and 95% confidence interval for the regression.* **(E)** *Density plots of pHER2 baseline semi-quantitative IHC, stratified by pathologic response in the final surgical specimens. Statistical significance determined using χ-squared test.*

Supplemental Figure 4

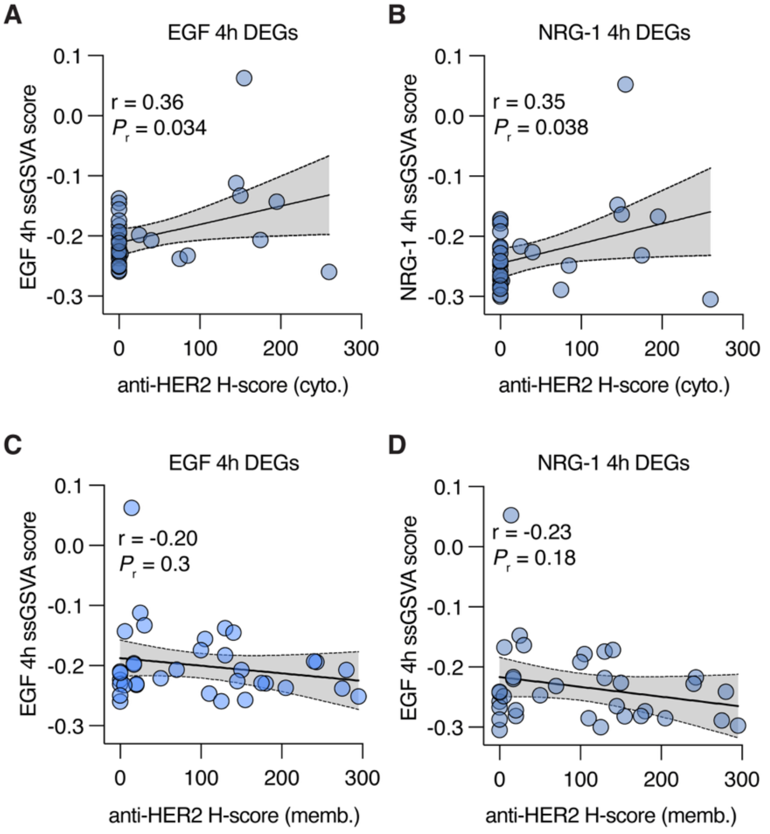

Supplemental Figure 4. Association between HER2 activation and HER2 IHC. *Scatter plots showing the association of matched foci (n = 35) of baseline H-scores (x-axis) of cytoplasmic* **(A–B)** *and membranous* **(C–D)** *HER2 IHC with single-sample GSVA scores of each transcriptome projected against the differentially-expressed geneset derived by stimulation of LNCaP cells for 4 h with EGF* **(A,C)** *or NRG-1* **(B,D)**. *Statistical significance determined using Pearson's correlation.*

Supplemental Figure 5

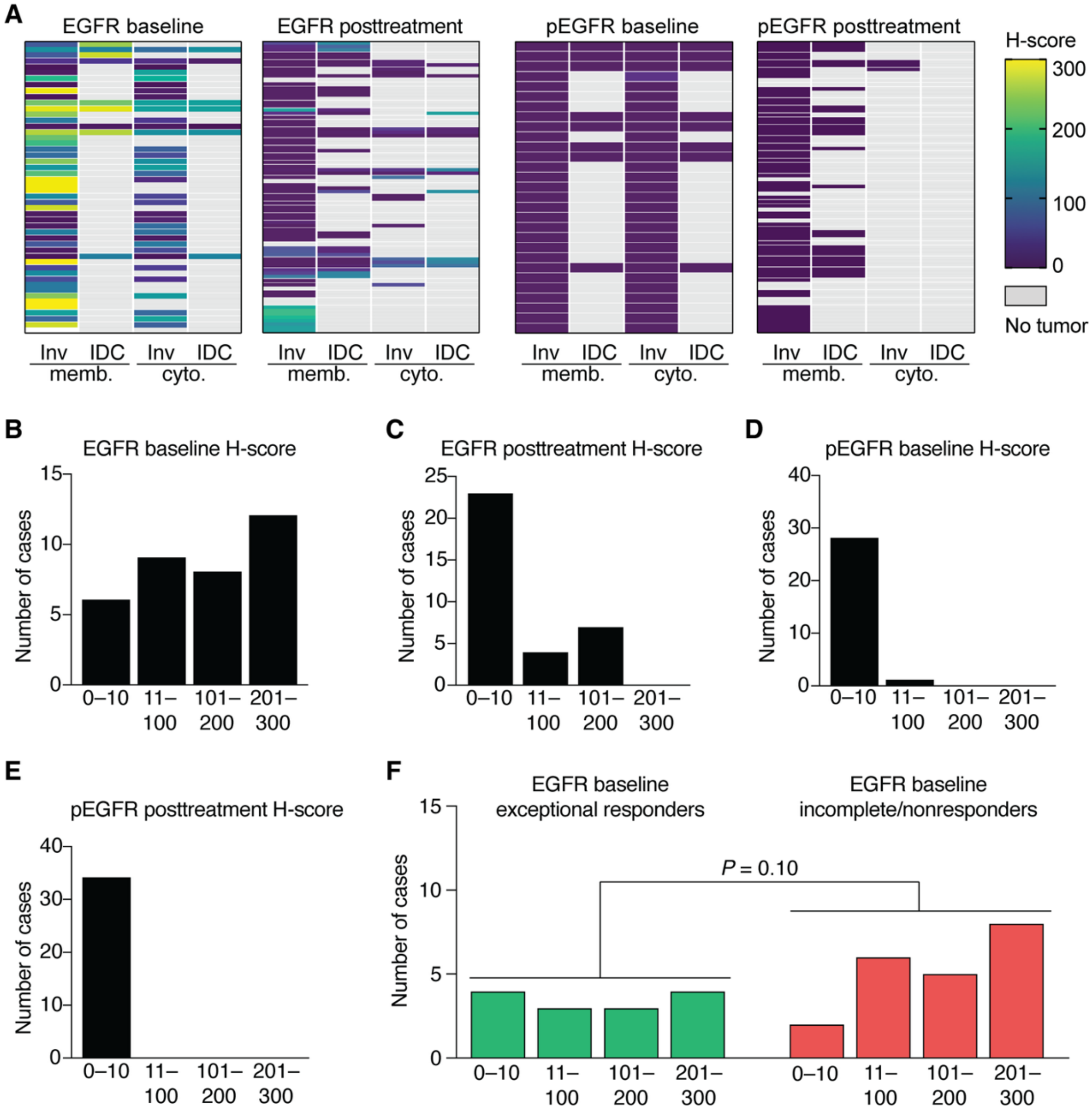

Supplemental Figure 5. Immunohistochemistry against EGFR and pEGFR. (A) *Heatmaps summarizing semi-quantitative analysis of anti-EGFR and anti-pEGFR IHC performed on entire sections of biopsies (n = 51 for EGFR; n = 30 for pEGFR) and posttreatment surgical specimens (n = 73 for EGFR; n = 67 for pEGFR). Rows are grouped by patient. Inv: invasive; IDC: intraductal carcinoma; memb: membranous; cyto: cytosolic.* **(B–E)** *Density plots summarizing the frequency distribution of IHC semi-quantitative analysis, per patient, of baseline biopsies (***B,D)** *or of posttreatment prostatectomy specimens* **(C,E)** *with antibodies against EGFR* **(B–C)** *and pEGFR* **(D–E)**. **(F)** *Density plots of EGFR baseline semi-quantitative IHC, stratified by pathologic response in the final surgical specimens. Statistical significance determined using χ-squared test.*

Supplemental Figure 6

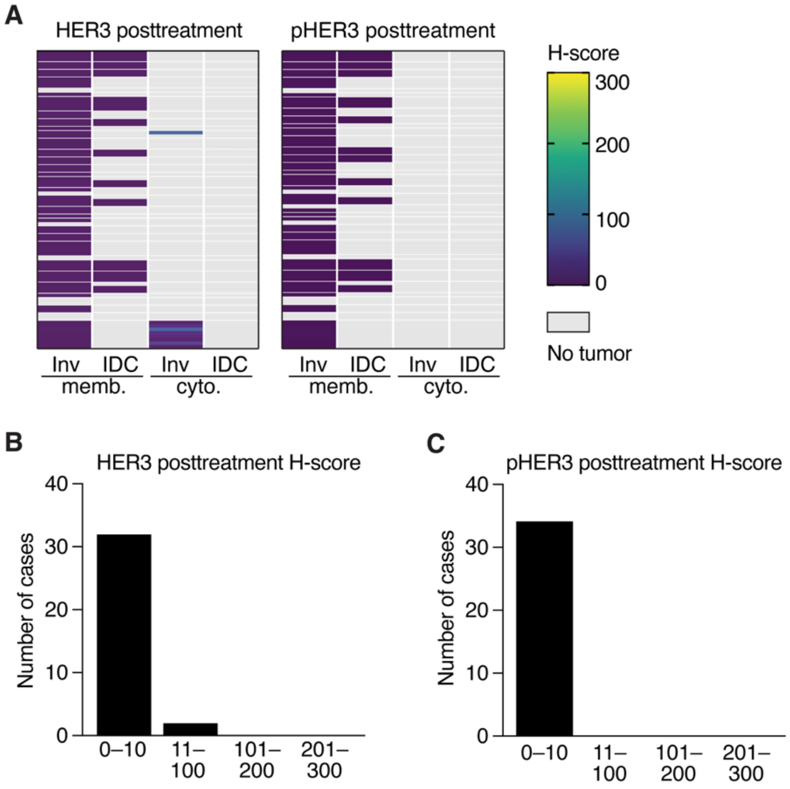

Supplemental Figure 6. Immunohistochemistry against HER3 and pHER3. (A) *Heatmaps summarizing semi-quantitative analysis of anti-HER3 and anti-pHER3 IHC performed on entire sections of posttreatment surgical specimens (n = 71 for HER3; n = 69 for pHER3). Inv: invasive; IDC: intraductal carcinoma; memb: membranous; cyto: cytosolic.* **(B–C)** *Density plots summarizing the frequency distribution of IHC semi-quantitative analysis, per patient, of posttreatment tumors with antibodies against HER3* **(B)** *and pHER3* **(C)**.

Supplemental Figure 7

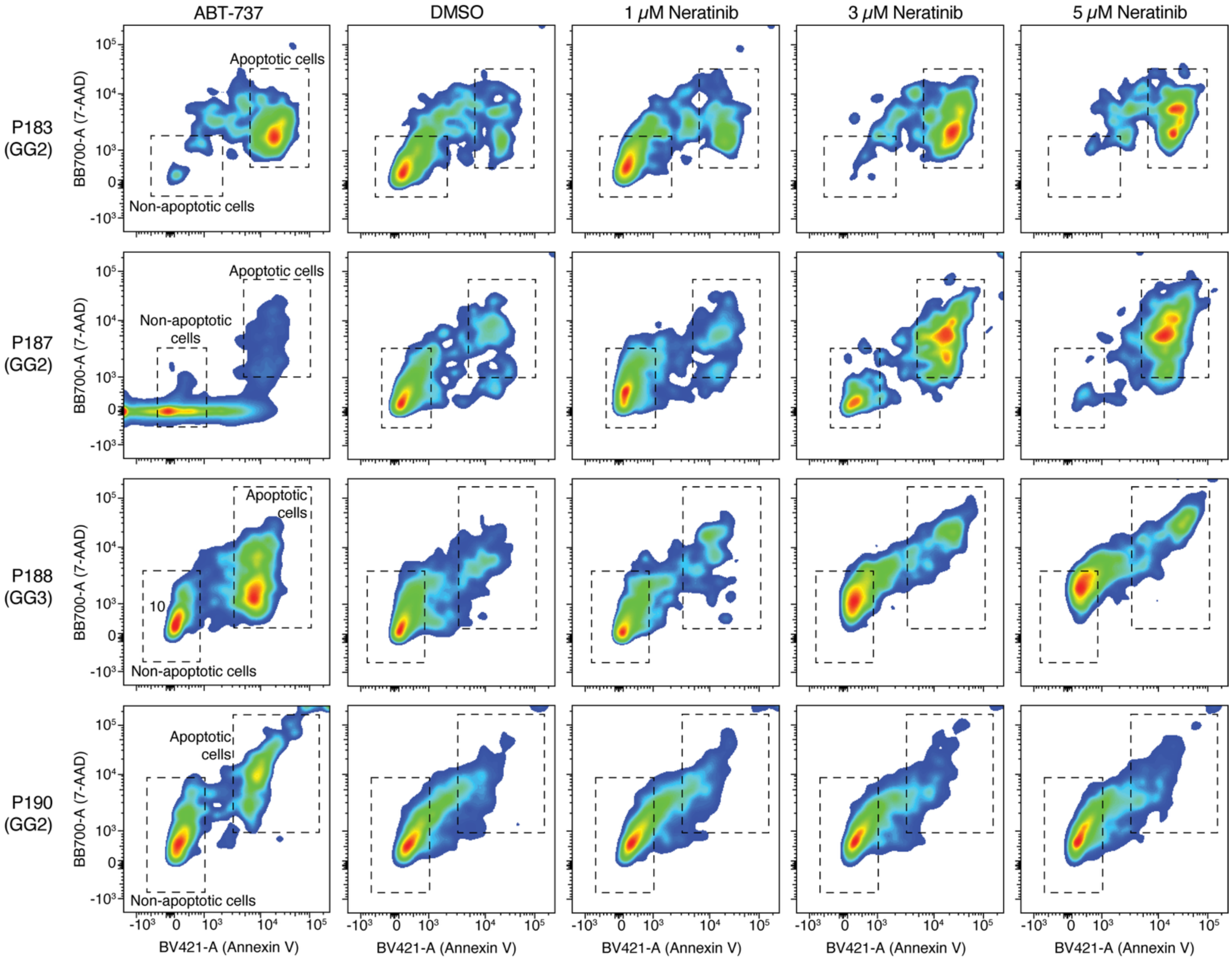

Supplemental Figure 7. Flow cytometry. *Flow cytometry of four human prostate cancer organoids treated with either 10 µM ABT-737 (to identify the gates for apoptotic and nonapoptotic cells for each model), or DMSO, 1 µM neratinib, 3 µM neratinib or 5 µM neratinib for 48 hours. The pathologic grade group of each of the organoid models (183, 187, 188 and 190) is also shown. The gates defined by ABT-737 treatment were used to identify the apoptotic and nonapoptotic cells.*

SUPPLEMENTAL REFERENCES

1. *Ku AT, Wilkinson S, and Sowalsky AG. Comparison of approaches to transcriptomic analysis in multi-sampled tumors. Briefings in bioinformatics. 2021;22(6).*
2. *Hoffman GE, and Roussos P. Dream: powerful differential expression analysis for repeated measures designs. Bioinformatics. 2021;37(2):192-201.*
3. *Wilkinson S, Ye H, Karzai F, Harmon SA, Terrigino NT, VanderWeele DJ, et al. Nascent Prostate Cancer Heterogeneity Drives Evolution and Resistance to Intense Hormonal Therapy. European urology. 2021.*
