# Supplementary figures and images for: Localized high-risk prostate cancer harbors an androgen receptor activity–low subpopulation susceptible to HER2 inhibition
Source: J Clin Invest. 2025 Sep 4;135(22):e189900. doi: 10.1172/JCI189900 (PMC12618079; doi:10.1172/JCI189900)

Full unedited blot for Supplemental Figure 1

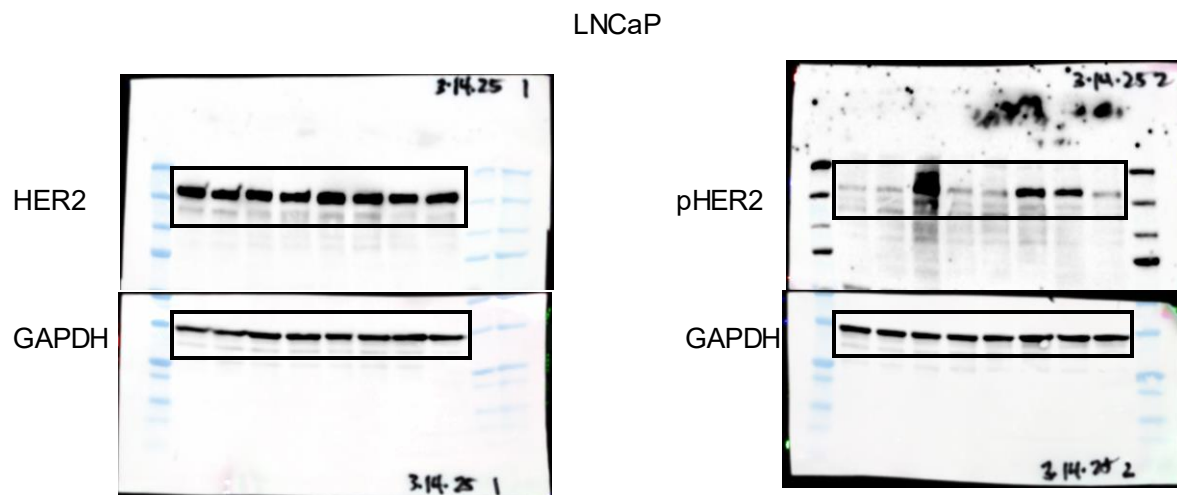

Full unedited blot for Figure 6

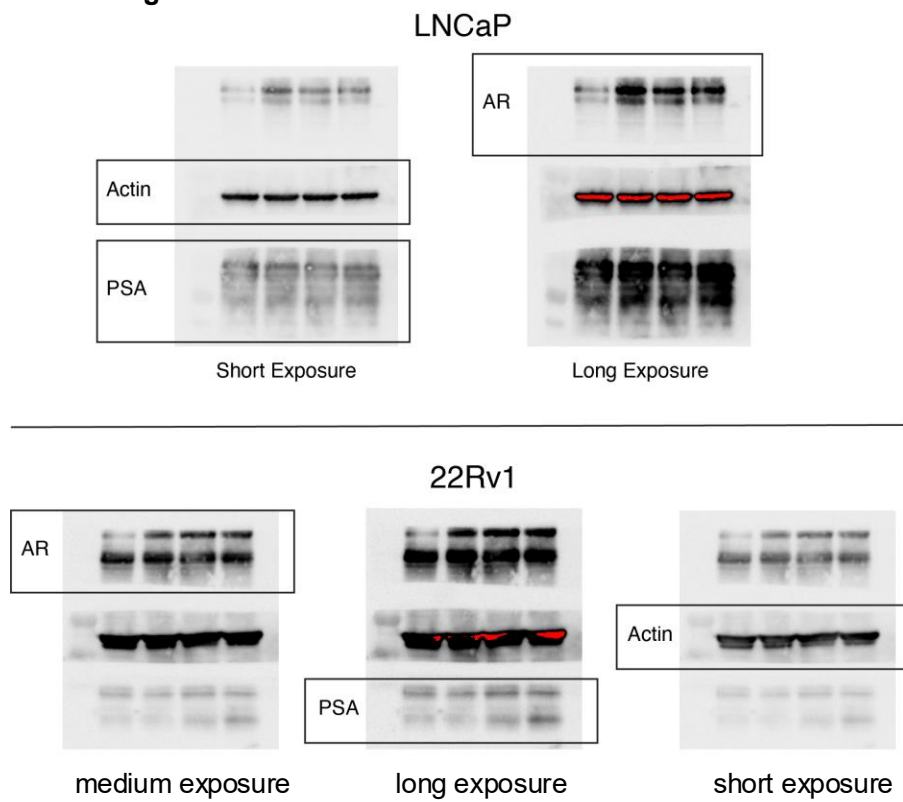

Supplement: Unedited blot and gel images [file jci-135-189900-s313.pdf]
